# Phytochemical-rich *Eucommia ulmoides* leaf extract extends healthspan in *Caenorhabditis elegans* via the *pmk-1*/p38 MAPK pathway and mitochondrial homeostasis

**DOI:** 10.3389/fnut.2025.1680518

**Published:** 2025-10-27

**Authors:** Jiaxu Zhang, Quhuan Ma, Jiaojiao Li, Xingwen Xie, Xiaofeng Shi

**Affiliations:** ^1^Institute of Pharmaceutical Research, Gansu Provincial Academic Institute for Medical Research, Lanzhou, China; ^2^College of Pharmacy, Gansu University of Chinese Medicine, Lanzhou, China; ^3^Clinical College of Chinese Medicine, Gansu University of Chinese Medicine, Lanzhou, China

**Keywords:** *Eucommia ulmoides* leaf, *Caenorhabditis elegans*, healthspan, mitochondrion, MAPK

## Abstract

**Objectives:**

As a medicinal and edible resource, *Eucommia ulmoides* leaf (EUL) shows considerable promise in facilitating healthy aging. However, its precise biological mechanisms remain unclear. This study aimed to investigate the anti-aging efficacy and underlying pathways of *Eucommia ulmoides* leaf extract (EULE) using the *Caenorhabditis elegans* (*C. elegans*) model.

**Methods:**

Phytochemical profiling of the extract was performed. *C. elegans* were supplemented with the extract, and the effects on lifespan, healthspan indicators (muscle deterioration, intestinal barrier function, mitochondrial homeostasis), and gene expression were evaluated via transcriptomic analysis.

**Results:**

Phytochemical analysis revealed that the extract is abundant in bioactive compounds such as flavonoids, iridoids, and lignans. Supplementation with the extract significantly prolonged the lifespan of *C. elegans* by up to 14.69% and improved healthspan by alleviating age-related muscle deterioration, preserving intestinal barrier function, and regulating mitochondrial homeostasis. Transcriptomic analysis identified the mitogen activated protein kinase (MAPK) signaling pathway as the primary mediator of these anti-aging benefits.

**Conclusions:**

These results provide robust evidence supporting the use of EUL as a natural dietary supplement to prolong healthspan, establishing a scientific rationale for its application in promoting healthy aging.

## 1 Introduction

Aging is a multifaceted biological phenomenon marked by the gradual deterioration of physiological systems and the impairment of homeostatic regulation. This process acts as a primary catalyst and significant risk factor for the onset of major chronic conditions, such as cancer, cardiovascular diseases, neurodegenerative disorders, and metabolic syndromes (1, 2). As global population aging accelerates, projections indicate that by 2050, the number of individuals aged 60 and over will reach 2.1 billion worldwide (3). Among this demographic, 75% of older adults suffer from at least one chronic disorder, while 43% face multimorbidity. Age-related diseases have imposed increasingly severe challenges on global public health systems and socioeconomic stability (4, 5). Consequently, identifying effective anti-aging intervention strategies is of paramount importance.

*Caenorhabditis elegans* (*C. elegans*) has emerged as a classic *in vivo* model for screening and evaluating anti-aging compounds due to its unique advantages, including a short lifespan, well-defined genetic background, and ease of genetic manipulation (6). Significantly, the mechanistic underpinnings of virtually all established lifespan-regulating signaling pathways, including the insulin/IGF-1 signaling (IIS) pathway, the mammalian target of rapamycin (mTOR) pathway, dietary restriction-mediated pathways, mitochondrial homeostasis, and autophagy were first identified and characterized in *C. elegans*. These pathways exhibit remarkable evolutionary conservation in mammals, positioning *C. elegans* as an invaluable model organism for the exploration of anti-aging bioactive compounds and the elucidation of their molecular mechanisms (7–9).

Mounting evidence indicates that bioactive compounds isolated from traditional Chinese medicine (TCM) demonstrate significant therapeutic potential for anti-aging interventions and age-associated pathologies, attributed to their extensive availability, structural diversity, favorable safety profiles, and pleiotropic mechanisms of action (10, 11). *Eucommia ulmoides* Oliv (EUO), a distinguished TCM herb, is a unique monotypic species increasingly recognized worldwide for its edible and health-promoting properties, making it a promising candidate for functional food development across diverse dietary cultures. Historically, its bark has been esteemed for its therapeutic properties in “tonifying the liver and kidneys, and fortifying bones and tendons” (12–14). Among its various components, the leaves are the most abundant and sustainably harvested. The EUL possess a long history of dietary use, with ancient texts documenting the edibility of their tender parts. Traditionally, their most widespread application is as a health-enhancing tea substitute, a practice highly popular in China, Japan, and other regions (15, 16). Beyond beverages, their use extends to cooking, porridge preparation, and the fortification of staple foods. Recognized for their robust safety profile, which is further confirmed by modern toxicological evaluations (17, 18), and substantiated health benefits, EUL have been officially listed in the “Medicinal and Edible Homology” catalog by the National Health Commission of China. This classification highlights the exceptional safety of EUL, supporting their potential as a promising candidate for long-term, natural anti-aging interventions (19–21). Modern pharmacological studies have revealed that EUL is rich in bioactive compounds such as chlorogenic acid, flavonoids, and iridoids (15, 22). Chlorogenic acid, a prominent phenolic constituent, exhibits potent antioxidant and hypoglycemic properties through free radical scavenging and the modulation of enzymatic pathways (23). Additionally, geniposidic acid, another principal compound, displays marked effectiveness in neutralizing free radicals, mediating anti-inflammatory processes, regulating apoptosis, and conferring neuroprotection (24–26). These findings underscore the considerable promise of EUL in mitigating age-related decline, thereby establishing a strong scientific basis for their application as a novel functional ingredient in foods aimed at promoting healthy aging.

This study employed *C. elegans* as a model organism to evaluate the effects of *Eucommia ulmoides* leaf extract (EULE) on lifespan and healthspan, aiming to elucidate its anti-aging activity and provide a scientific foundation for its application in functional foods and natural anti-aging therapeutics.

## 2 Materials and methods

### 2.1 Reagents and materials

The EUL were purchased from Zhejiang Zhenyuantang Chinese Herbal Pieces Co., Ltd. (Jinhua, Zhejiang, China; Batch No. 20230201). The plant material was authenticated by the supplier in compliance with the quality standards of the *Chinese Pharmacopoeia* (2020 edition). A reference sample has been retained in the specimen storage room at Gansu Provincial Academic Institute for Medical Research. Assay kits for catalase (CAT), superoxide dismutase (SOD), reactive oxygen species (ROS), malondialdehyde (MDA), reduced glutathione (GSH), hydroxyproline (HYP), and mitochondrial membrane potential (MMP) (TMRE-based) were obtained from Beijing Solarbio Science & Technology Co., Ltd. (Beijing, China). The ATP quantification kit was provided by Shanghai Jianglai Biological Technology Co., Ltd. (Shanghai, China). 5-Fluoro-2′-deoxyuridine (5-FUdR) and levamisole hydrochloride were purchased from Shanghai Macklin Biochemical Co., Ltd. (Shanghai, China). Unless specified otherwise, all chemicals and reagents were of analytical grade and acquired from commercial vendors.

### 2.2 Strains and culture conditions

All *C. elegans* strains utilized in this investigation were sourced from the Caenorhabditis Genetics Center at the University of Minnesota, USA. The strains included the wild-type N2 and the following transgenic lines: CF1553 (muIs84) [sod-3p::GFP + rol-6 (su1006)], RW1596 (stEx30) [myo-3p::GFP::myo-3 + rol-6 (su1006)], PD4251 (ccIs4251) [myo-3p::GFP::LacZ::NLS + myo-3p::mitochondrial GFP + dpy-20 (+)] I, CB7272 (ccIs4251) [myo-3p::GFP::LacZ::NLS + myo-3p::mitochondrial GFP + dpy-20 (+)] I, and KU25 (*pmk-1 (km25)* IV). All strains were maintained according to established nematode husbandry protocols (27). Briefly, animals were cultured at 20 °C on nematode growth medium (NGM) plates seeded with *Escherichia* coli OP50 (*E*. coli OP50) as a food source. Synchronized populations were generated by isolating eggs from gravid adults using hypochlorite treatment. The hypochlorite solution was freshly prepared by combining 5 M NaOH, 5% sodium hypochlorite, and M9 buffer in a 1:2:7 volume ratio. EULE was dissolved in ultrapure water to create a stock solution, which was subsequently mixed with *E. coli* OP50 at a 1:19 (v/v) ratio. The resulting mixture was evenly distributed onto NGM plates for experimental assays.

### 2.3 Preparation and chemical composition analysis of EULE

Approximately 200.00 g of EUL was mixed with ultrapure water using a solid-to-liquid ratio of 1:15. Following a 1-h soaking period, reflux extraction was performed for 1 h, and the extraction was repeated twice. The resulting filtrates were combined, concentrated under reduced pressure at 60 °C, and the final extract was obtained (yield: 28.37%, w/w). Subsequently, the extract was freeze-dried under vacuum and stored for subsequent use.

The extract's chemical profile was characterized using ultra-high-performance liquid chromatography coupled with quadrupole-exactive Orbitrap mass spectrometry (UHPLC-Q-Exactive Orbitrap MS). Chromatographic separation was achieved on a Thermo Scientific™ U3000 UHPLC system equipped with a Hypersil GOLD C_18_ column (100 × 2.1 mm, 1.9 μm). The injection volume was 2 μL, with the column maintained at 40 °C and a flow rate of 0.3 mL/min. The mobile phases consisted of 0.1% formic acid in water (A) and 0.1% formic acid in acetonitrile (B), with a programmed gradient: 0–4 min, 5%−8% B; 4–10 min, 8%−12% B; 10–15 min, 12%−20% B; 15–22 min, 20%−35% B; 22–28 min, 35%−55% B; 28–30 min, 55%−97% B; 30–33 min, 97%−100% B; 33–35 min, 100% B.

Mass spectrometric analysis was performed on a Thermo Scientific Q-Exactive Orbitrap MS, utilizing an electrospray ionization (ESI) source in both positive (ESI+) and negative (ESI–) ion modes. The ion transfer capillary temperature was set at 320 °C, with spray voltages of 3,500 V (ESI+) and 3,000 V (ESI–). Nitrogen was used as both sheath and auxiliary gases, with flow rates of 30/10 arb (ESI+) and 35/16 arb (ESI–), respectively. Data acquisition employed full MS/dd-MS^2^ (Top4) mode, with a primary scan range of 120–1,000 Da. The resolution was set at 35,000 FWHM for full MS and 17,500 FWHM for MS^2^. Automatic gain control (AGC) targets were 5 × 106 for both MS and MS^2^, with maximum injection times of 100 ms (MS) and 25 ms (MS^2^). High-energy collision-induced dissociation (HCD) was used for MS^2^ fragmentation, with a collision energy of 20 eV, an isolation window of 0.4 Da, and a dynamic exclusion duration of 4.5 s.

### 2.4 Impact of EULE on the survival and longevity of *C. elegans*

To assess the safety profile and longevity-promoting potential of EULE in *C. elegans*, we initially performed acute toxicity assays. Synchronized N2 *C. elegans* at the L4 larval stage were allocated to 96-well plates at a density of ten worms per well and maintained in S-complete liquid medium supplemented with varying concentrations of EULE (0–8 mg/mL) and *E. coli* OP50. Following a 24-h incubation at 20 °C, survival rates were quantified to establish the non-toxic concentration range. Lifespan assays were conducted following established protocols (28), with minor modifications. Briefly, 50 L4-stage worms to NGM agar plates containing EULE at the established safe concentrations (1, 2, and 4 mg/mL) in the presence of 50 μM FUdR. Survival was monitored daily from day 0 (the day of transfer), with worms being relocated to fresh plates as needed until all individuals had expired. Death was operationally defined as a lack of response to gentle stimulation with a platinum wire. Worms that died due to extraneous factors or vulval rupture were excluded from the dataset. The mean and maximum lifespans for each experimental group were then calculated.

### 2.5 Effects of EULE on the proliferation of *E. coli* OP50 and feeding behavior of *C. elegans*

**Figure 1:**
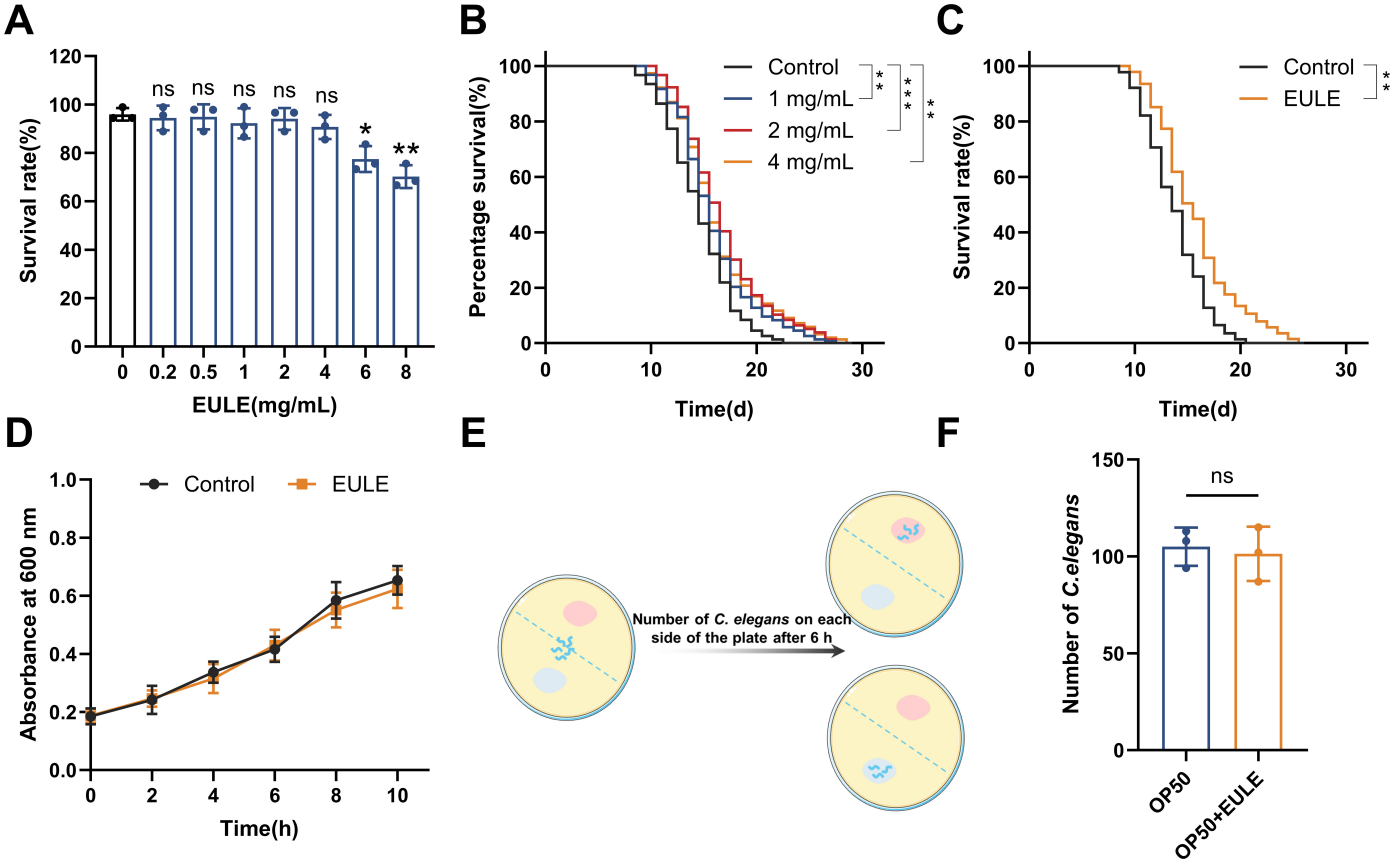
EULE extends the lifespan of *C. elegans* independently of the bacterial diet *E. coli* OP50. **(A)** Survival rate of wild-type N2 *C. elegans* after 24-h treatment with varying concentrations of EULE (0, 0.2, 0.5, 1, 2, 4, 6, 8 mg/mL). **(B)** Survival curves of wild-type N2 *C. elegans* treated with different EULE concentrations (1, 2, 4 mg/mL) at 20 °C. **(C)** Survival curves of wild-type N2 *C. elegans* fed with heat-inactivated (65 °C, 30 min) *E. coli* OP50 or *E. coli* OP50 supplemented with EULE. **(D)** Optical density (OD 600) values of *E. coli* OP50 cultured in LB liquid medium with or without EULE at different time points. **(E)** Schematic diagram of the food preference assay. **(F)** Distribution of wild-type N2 *C. elegans* between *E. coli* OP50-containing regions with or without EULE supplementation. All statistical analyses were performed using unpaired two-tailed Student's *t-*test. *p < 0.05, **p < 0.01, ***p < 0.001.

To assess the impact of EULE on *E. coli* OP50 proliferation and the feeding behavior of *C. elegans*, 50 μL of EULE (final concentration: 1 mg/mL) or sterile water (as the control) was introduced into 150 μL of *E. coli* OP50 suspension within a 96-well microplate. Blank wells containing only the extract in medium were included to correct for any absorbance interference due to the inherent coloration of the extract. The microplate was incubated at 37 °C, and the optical density (OD_600_) was recorded at 0, 1, 2, 4, 6, 8, and 10 hours post-inoculation (29). Subsequently, a food preference assay was performed by dispensing 80 μL of standard *E. coli* OP50 suspension and 80 μL of the *E. coli* OP50-EULE mixture onto opposite sides of a 90-mm NGM agar plate. Approximately 200 synchronized L4-stage N2 *C. elegans* were placed equidistant from both food sources at the center of the plate and incubated at 20 °C for 6 h. The distribution of worms in each food region was quantified and statistically analyzed (Figure 1E) (30).

### 2.6 Measurement of reproductive rate

To assess reproductive output, ten synchronized N2 *C. elegans* at the L4 larval stage were individually seeded onto NGM plates devoid of FUdR, each exposed to varying concentrations. Worms were transferred to fresh plates every 24 hours until the cessation of oviposition. The number of viable progeny was quantified 48 hours post-transfer, enabling calculation of both daily and cumulative fecundity per individual (31).

### 2.7 Stress resistance assays

Stress resistance assays were conducted following established protocols (32), with minor modifications. Briefly, oxidative stress assay: After a 5-day incubation with EULE, *C. elegans* were transferred to NGM plates supplemented with 300 μM juglone (ethanol as solvent). Survival rates were monitored at 2-h intervals until complete mortality was observed.

Thermal Stress Assay: Following a 5-day exposure to EULE, *C. elegans* were subjected to heat stress at 35 °C. Viability was assessed every 2 h until all specimens had expired.

### 2.8 Assessment of aging phenotypes in *C. elegans*

To investigate the impact of EULE on age-associated phenotypes in *C. elegans*, we quantified pharyngeal pumping rate and body length at days 5, 10, and 15 of cultivation, and body bend frequency was quantified in M9 buffer (33, 34). Furthermore, locomotor function was assessed and classified as Grade A (normal sinusoidal locomotion), Grade B (reduced or irregular movement), or Grade C (movement restricted to head or tail upon tactile stimulation). Intestinal barrier integrity was evaluated in 15-day-old worms following EULE treatment from the L4 larval stage (35). Briefly, worms were collected and washed three times with M9 buffer, then incubated with 5% brilliant blue (w/v in M9 buffer) supplemented with 10 μL *E. coli* OP50 for 5 h at 20 °C. After washing to remove excess dye, worms were anesthetized with levamisole and mounted on 2% agarose pads for microscopic examination. A compromised intestinal barrier was identified by the leakage of dye from the intestinal lumen into the body cavity. Furthermore, morphological alterations in mid-body muscle fibers were examined in the RW1596 strain at days 5 and 13. Damage to muscle fibers was identified by the presence of obvious fractures, loss, or disordered arrangement of the body wall muscle fibers (36).

### 2.9 Detection of spontaneous lipofuscin fluorescence in *C. elegans*

Synchronized N2 *C. elegans* at the L4 stage were randomly transferred to NGM plates containing different concentrations of the test compounds, with 50 worms per group. All worms were cultured in a constant-temperature incubator at 20 °C. To prevent starvation and maintain constant drug concentrations, the worms were transferred to fresh plates containing the corresponding drug concentrations every other day. After 10 days of culture, the worms from each group were collected into 2 mL centrifuge tubes using M9 buffer and washed three times to remove residual *E.coli* OP50 bacteria and drug compounds. The worms were then anesthetized with 25 mM levamisole hydrochloride and transferred to a 2% agarose-coated slide for immobilization (*n* ≥ 30 worms per group). Observation and imaging were performed under a fluorescence microscope. The acquired images were processed and analyzed using Image J software (34).

### 2.10 Assessment of ROS levels and MMP

Following a 10-day incubation of *C. elegans* in EULE, approximately 50 worms from each group wormswere harvested into 2 mL microcentrifuge tubes and subjected to three washes with M9 buffer. Post-centrifugation, the supernatant was removed. For ROS quantification, 1.5 mL of DCFH-DA dye (diluted in M9 buffer) was administered at a final concentration of 50 μM. For assessment of MMP, 1.5 mL of TMRE staining solution (diluted in M9 buffer) was applied at a final concentration of 5 × . All samples were shielded from light using aluminum foil and incubated at 20 °C for 4 h. Following incubation, worms were washed three times with M9 buffer to eliminate residual dye (37, 38). Subsequently, at least 30 worms per group were randomly selected, anesthetized with levamisole hydrochloride and mounted on 2% agarose pads. Fluorescence microscopy was employed for visualization and image acquisition, and the resulting images were processed and quantitatively analyzed using ImageJ software.

### 2.11 Assessment of antioxidant enzyme activities, MDA, HYP, and ATP levels

Approximately 2,000 *C. elegans* were incubated with EUEL for 5 and 10 days, respectively, and subsequently harvested. The worms were rinsed three times with M9 buffer, after which extraction buffer was added and the samples were homogenized by cryogenic grinding at 4 °C. The resulting homogenate was centrifuged at 12,000 rpm for 10 min at 4 °C, and the supernatant was collected for downstream analyses (39). Total protein concentration in the supernatant was determined using a BCA protein assay kit. The supernatant was then utilized to assess SOD, CAT, MDA, GSH, HYP, and ATP levels in the worms, according to the protocols provided by the respective assay kit manufacturers.

### 2.12 Quantification of SOD-3 expression, mitochondrial content, and respiratory chain complex levels

Following cultivation of *C. elegans* strains on EULE medium for 5, 10, and 13 days, respectively, worms were harvested into 2 mL microcentrifuge tubes and subjected to three washes with M9 buffer. To assess specific physiological indicators, three distinct transgenic strains were employed. The strain CF1553, which expresses a SOD-3::GFP fusion protein, was utilized to determine the expression level of the antioxidant enzyme superoxide dismutase-3 by detecting the expression of GFP (40). The strain PD4251, carrying a myo-3p::GFP reporter that expresses GFP in all body wall muscle cells, was used to visualize mitochondria and quantify changes in mitochondrial content (41). The strain CB7272 was employed to simultaneously measure the levels of mitochondrial respiratory chain complexes; in this strain, complexes I, II, and III are tagged with GFP, while complexes IV and V are tagged with RFP (42). The specimens were then anesthetized with levamisole hydrochloride and mounted on 2% agarose pads for immobilization. Fluorescence microscopy was utilized for visualization and image acquisition, and fluorescence intensity was quantitatively analyzed using ImageJ software.

### 2.13 Transcriptome sequencing

For transcriptomic analysis, *C. elegans* were collected on day 5 of adulthood. This time point was strategically chosen to identify the initial, upstream molecular mechanisms underlying the pro-longevity effects of EULE, before the accumulation of extensive and irreversible age-related damage. To obtain the samples, approximately 5,000 individuals per group were washed three times with M9 buffer to remove residual *E. coli* OP50. The worm samples were then rapidly frozen in liquid nitrogen and stored at−80 °C (43). Total RNA was isolated using the TRIzol reagent. Subsequent RNA purification, cDNA synthesis, library construction, and high-throughput sequencing were carried out by Shanghai Personal Biotechnology Co., Ltd. (Personal Biotechnology, Shanghai, China).

Following sequencing, raw reads underwent quality assessment and filtering with FASTP to eliminate adapter contamination and low-quality sequences (Q-score <20). High-quality reads were mapped to the *C. elegans* reference genome (http://metazoa.ensembl.org/Caenorhabditis_elegans/Info/Index) using HISAT2. Differential gene expression analysis was performed, defining differentially expressed genes (DEGs) as those with |log2 (fold change)| > 1 and *p*-value <0.05. Functional enrichment analyses, including Gene Ontology (GO) and Kyoto Encyclopedia of Genes and Genomes (KEGG) pathway analyses, were conducted utilizing genome annotation resources.

### 2.14 RT-qPCR

Wild-type N2 *C. elegans* were cultured in EULE-supplemented medium for 5 days, after which the worms were harvested and total RNA was isolated utilizing the Total RNA Extractor (Trizol) Kit (Sangon Biotech, Shanghai, China). First-strand cDNA synthesis was carried out with the Swe-Script RT First Strand cDNA Synthesis Kit (Servicebio, Wuhan, China). Quantitative real-time PCR (RT-qPCR) was subsequently conducted on a CFX 96 Real-Time PCR System (Bio-Rad, Hercules, CA, USA) using the SGExcel FastSYBR Mixture (Sangon Biotech, Shanghai, China). The *act-1* gene served as the endogenous control, and relative transcript levels of the genes of interest were determined via the 2^−Δ*ΔCt*^ method (34, 39). Primer sequences employed in this study are provided in Supplementary Table S1.

### 2.15 Statistical analysis

Statistical analyses were performed using GraphPad Prism 10.0 software, with all experiments repeated at least three times. Data are presented as mean ± standard deviation (SD). Survival curves were constructed using the Kaplan-Meier method, and statistical significance was assessed by the log-rank test. Unless otherwise specified, the Student's *t-*test was applied for other comparisons. A *p*-value > 0.05 was considered not statistically significant (ns), whereas *p*-values <0.05 were deemed statistically significant (^*^p < 0.05, ^**^p < 0.01, ^***^p < 0.001).

## 3 Results

### 3.1 Composition analysis of EULE

**Figure 2:**
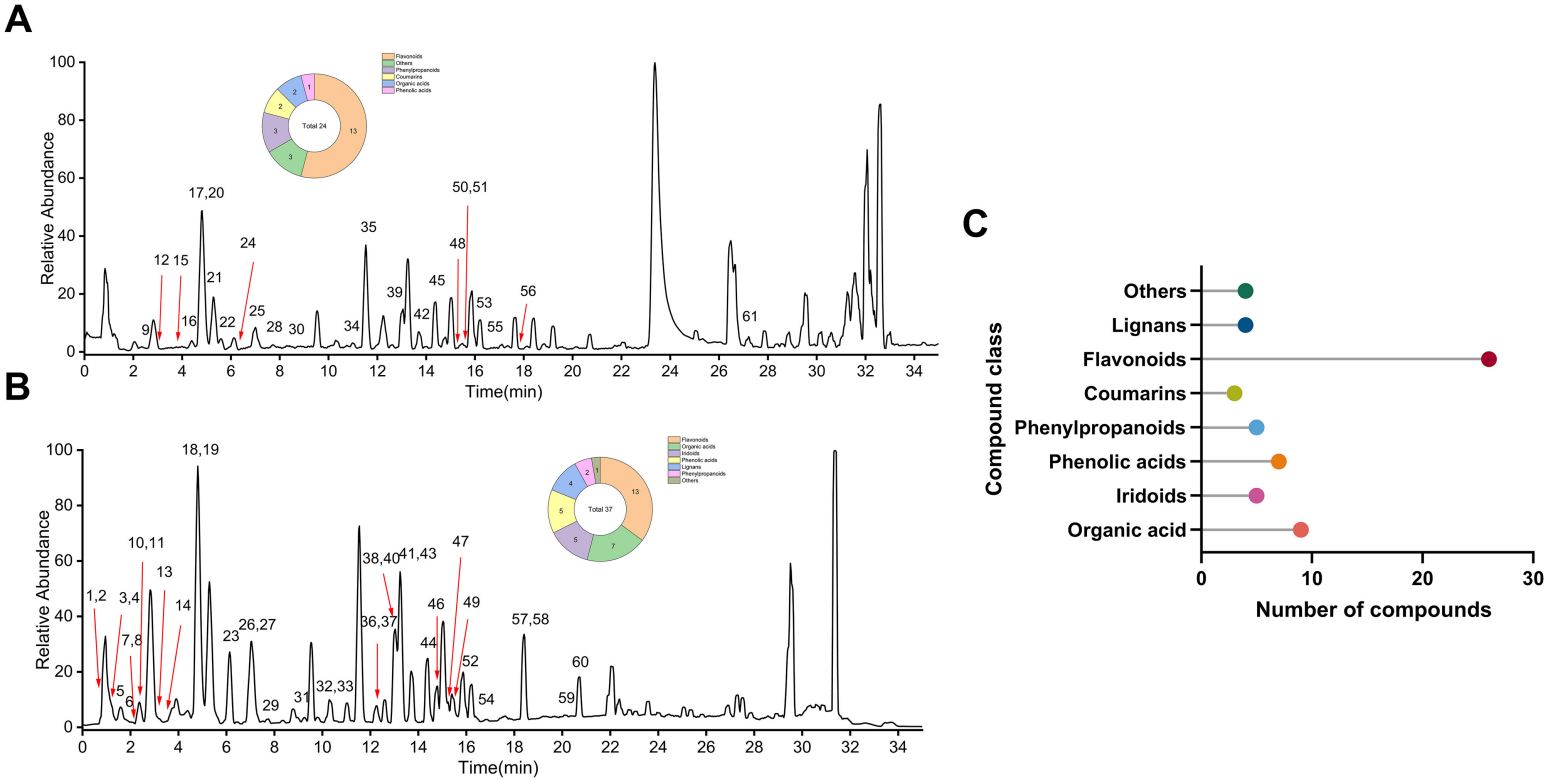
Chemical profiling of EULE. **(A)** Base peak chromatogram in positive ion mode. **(B)** Base peak chromatogram in negative ion mode. **(C)** Classification and number of the 63 compounds identified in EULE.

The phytochemical composition of EULE was elucidated using UPLC-Q-Exactive Orbitrap mass spectrometry. To ensure extensive metabolomic coverage, chromatographic analyses were performed under both positive and negative electrospray ionization conditions (Figure 2). By systematically evaluating mass-to-charge (*m/z*) ratios and MS/MS fragmentation spectra, a total of 61 unique metabolites were confidently identified in EULE, as summarized in Supplementary Table S2.

These metabolites were categorized into eight principal classes. Flavonoids constituted the predominant group (*N* = 26), detected in both ionization modes. The majority of organic acids (*N* = 9), phenolic acids (*N* = 6), iridoids (*N* = 5), and lignans (*N* = 4) were primarily observed in the negative ion mode. Conversely, all two coumarins were exclusively detected in the positive ion mode, which also facilitated the identification of five phenylpropanoids and four additional compounds.

### 3.2 EULE prolongs the lifespan of *C. elegans*

Before conducting lifespan assays, the non-toxic concentration range of EULE for *C. elegans* was established. Data indicated that EULE at 0.2–4 mg/mL did not adversely affect *C. elegans* viability. In contrast, exposure to 6 or 8 mg/mL EULE resulted in a significant reduction in 24-h survival rates to 77.47 ± 4.38% (*p* < 0.05) and 70.21 ± 3.84% (*p* < 0.01), respectively (Figure 1A). Consequently, subsequent experiments utilized EULE within the established safe concentration range. To assess the impact of EULE on *C. elegans* longevity, worms were treated with 1, 2, and 4 mg/mL EULE at 20 °C. As presented in Supplementary Table S3 and Figure 1B, all treatment groups within the 1–4 mg/mL range exhibited a statistically significant extension in mean lifespan compared to controls, reaching 16.29 ± 0.39, 17.20 ± 0.26, and 16.78 ± 0.27 days, respectively (control: 14.99 ± 0.17 days). Furthermore, the maximum lifespan of EULE-treated worms was markedly increased to 27, 27, and 28 days, relative to 22 days in the control group. Notably, the 2 mg/mL EULE treatment produced the most substantial effect, enhancing mean lifespan by 14.69% (*p* < 0.001) and maximum lifespan by 22.72% compared to controls. These findings demonstrate that EULE significantly promotes lifespan extension in *C. elegans* at concentrations of 1–4 mg/mL. Based on the pronounced efficacy observed at 2 mg/mL, this concentration was selected for subsequent mechanistic studies on the anti-aging properties of EULE.

### 3.3 EULE prolongs the lifespan of *C. elegans* independently of *E. coli* OP50

Prior research has established that *E. coli* OP50 can indirectly modulate *C. elegans* lifespan via the biotransformation of pharmacological agents. To elucidate whether the pro-longevity effect of EULE is attributable to bioactive metabolites produced through bacterial metabolism, EULE was administered in conjunction with heat-inactivated *E. coli* OP50 (65 °C, 30 min) during lifespan assays. Relative to controls, *C. elegans* exposed to heat-inactivated *E. coli* OP50 supplemented with EULE demonstrated a mean lifespan of 16.23 ± 0.32 days, compared to 14.26 ± 0.33 days in the control group, corresponding to a 13.81% increase (*p* < 0.01). The maximum lifespan observed in the EULE-treated group was 26 days, surpassing the 21-day maximum in controls (Figure 1C). These findings indicate that the lifespan-extending properties of EULE are independent of metabolic processing by *E. coli* OP50 and are instead attributable to the direct bioactivity of the extract's inherent constituents.

Given that *E. coli* OP50 serves as the principal dietary source for *C. elegans*, its growth dynamics could potentially impact worm physiology and confound longevity assessments. To determine whether EULE exerts indirect effects on lifespan by modulating bacterial proliferation, its influence on *E. coli* OP50 growth was evaluated. Measurement of optical density at 600 nm revealed no statistically significant difference between EULE-treated and untreated *E. coli* OP50 cultures (*p* > 0.05) (Figure 1D), indicating that EULE does not affect bacterial proliferation. This further substantiates that the observed biological effects are not mediated via alterations in bacterial metabolism, thereby excluding confounding effects from disrupted bacterial growth.

Food preference assays revealed no significant difference in the spatial distribution of *C. elegans* between the two bacterial lawns (*p* > 0.05) (Figure 1F), suggesting an absence of aversion to EULE. Feeding behavior appeared random with respect to the two food sources, thereby ruling out dietary preference as a confounding variable.

### 3.4 EULE enhances the health status of *C. elegans*

**Figure 3:**
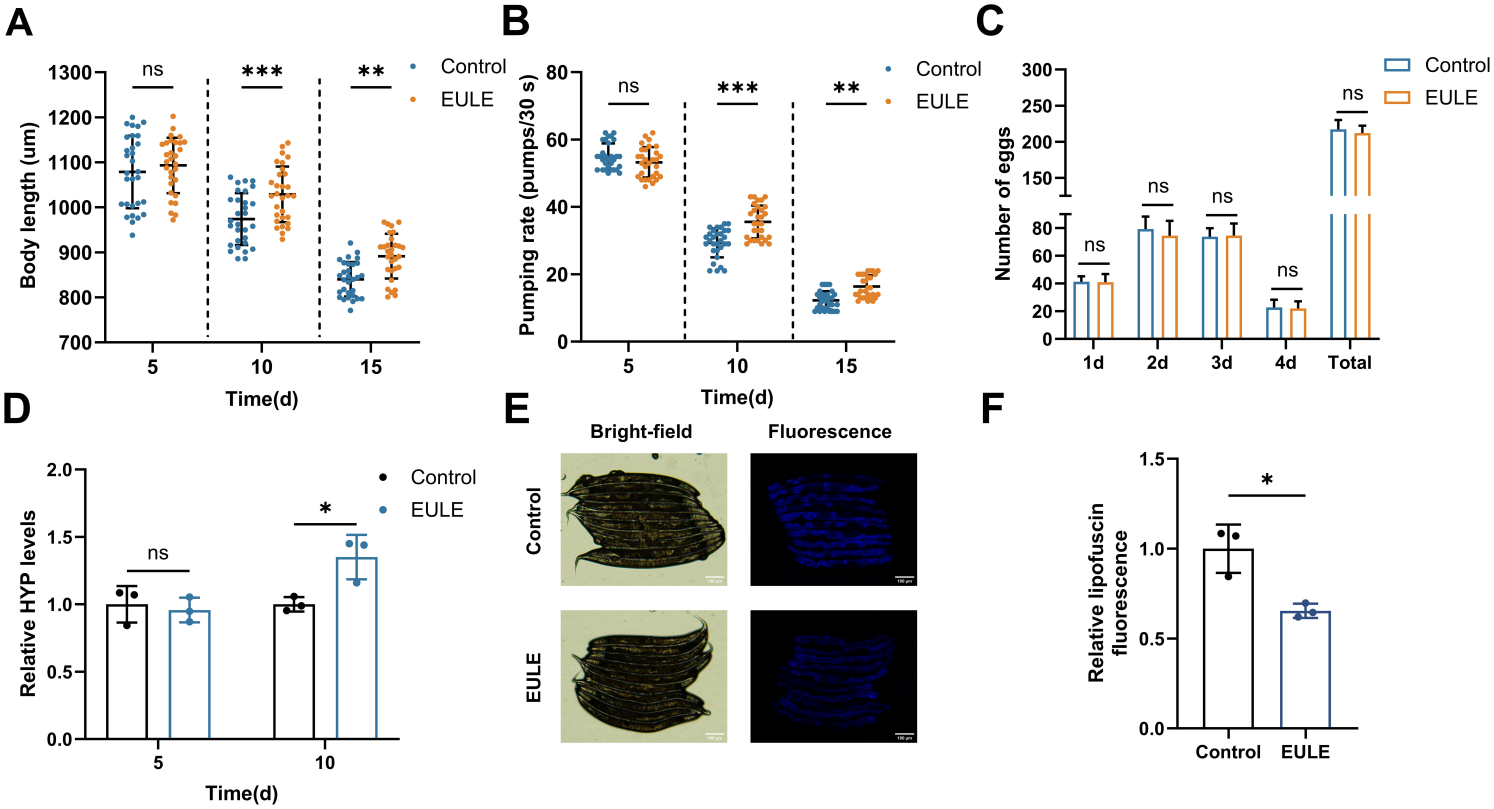
EULE significantly enhances the health status of *C. elegans*. **(A)** Changes in body length of wild-type N2 *C. elegans* after 5, 10, and 15 days of EULE treatment. **(B)** Pharyngeal pumping frequency within 30 seconds in wild-type N2 *C. elegans* after 5, 10, and 15 days of EULE treatment. **(C)** Comparison of egg-laying capacity between the control and EULE-treated *C. elegans*, including daily egg production during the reproductive period and total egg production. **(D)** Relative HYP content in wild-type N2 *C. elegans* after 5 and 10 days of EULE treatment. **(E)** Representative bright-field and fluorescence images of wild-type N2 *C. elegans* after 10 days of EULE treatment. Scale bar: 100 μm. **(F)** Relative intensity of intestinal lipofuscin autofluorescence in wild-type N2 *C. elegans* following 10 days of EULE treatment. All statistical analyses were performed using unpaired two-tailed Student's *t-*test. *p < 0.05, **p < 0.01, ***p < 0.001.

To assess the salutary effects of EULE on the physiological health of *C. elegans*, this investigation evaluated the influence of EULE on body length, pharyngeal pumping activity, and reproductive rate. Body length, an indicator of *C. elegans* morphology that diminishes with senescence, was measured at multiple time points. At day 5, no statistically significant difference was detected between the control and EULE-treated group (*p* > 0.05). By day 10, however, the EULE-treated group exhibited a significantly greater mean body length (1,028.77 ± 60.62 μm) compared to controls (974.03 ± 56.69 μm) (*p* < 0.01). At day 15, the control group experienced a 22.1% reduction in body length relative to day 5, whereas the EULE group showed only an 18.44% decrease, underscoring a more substantial preservation of morphology (Figure 3A).

EULE supplementation also significantly ameliorated the age-related decline in pharyngeal pumping rate. In advanced age, the EULE group maintained markedly higher pharyngeal pumping frequencies than controls, with increases of 21.14% (*p* < 0.001) and 33.79% (*p* < 0.001) at days 10 and 15, respectively, indicative of enhanced feeding efficiency in senescent worms (Figure 3B). Given that longevity-promoting interventions can sometimes impair fecundity, the impact of EULE on reproductive performance was also evaluated. No significant differences (*p* > 0.05) were observed in either peak daily egg-laying or total brood size (control: 217.20 ± 12.26 eggs; EULE: 212.20 ± 9.98 eggs) between groups, indicating that EULE did not compromise reproductive fitness. Collectively, these data demonstrate that EULE significantly mitigates age-associated declines in somatic length and pharyngeal pumping, extends mean lifespan, and preserves reproductive capacity in *C. elegans* (Figure 3C).

Collagen, a pivotal biomarker for organismal skin aging, was assessed via quantification of its hallmark component, HYP. To elucidate the anti-aging effects of EULE, HYP content was measured in *C. elegans*. During early aging, no significant intergroup differences in HYP levels were detected (*p* > 0.05). By day 10, however, the EULE group exhibited a 34.97% elevation in HYP content relative to controls (*p* < 0.05), indicating that EULE intervention effectively attenuated collagen depletion during aging (Figure 3D).

### 3.5 EULE decreases lipofuscin accumulation in *C. elegans*

Lipofuscin, an end-stage byproduct resulting from impaired lysosome-dependent autophagic flux, is predominantly composed of non-degradable, oxidized lipid-protein cross-linked aggregates. Its intrinsic autofluorescence facilitates quantitative assessment of lysosomal storage burden associated with organismal aging. In this investigation, intestinal lipofuscin fluorescence in *C. elegans* was quantified at day 10 of adulthood. The EULE-treated group demonstrated a 34.57% decrease in fluorescence intensity relative to controls (*p* < 0.05) (Figures 3E, F). These findings indicate that EULE significantly attenuates intestinal lipofuscin accumulation in *C. elegans*, thereby mitigating *in vivo* metabolic waste deposition.

### 3.6 EULE delays aging by preserving intestinal barrier integrity and improving muscle status in *C. elegans*

**Figure 4:**
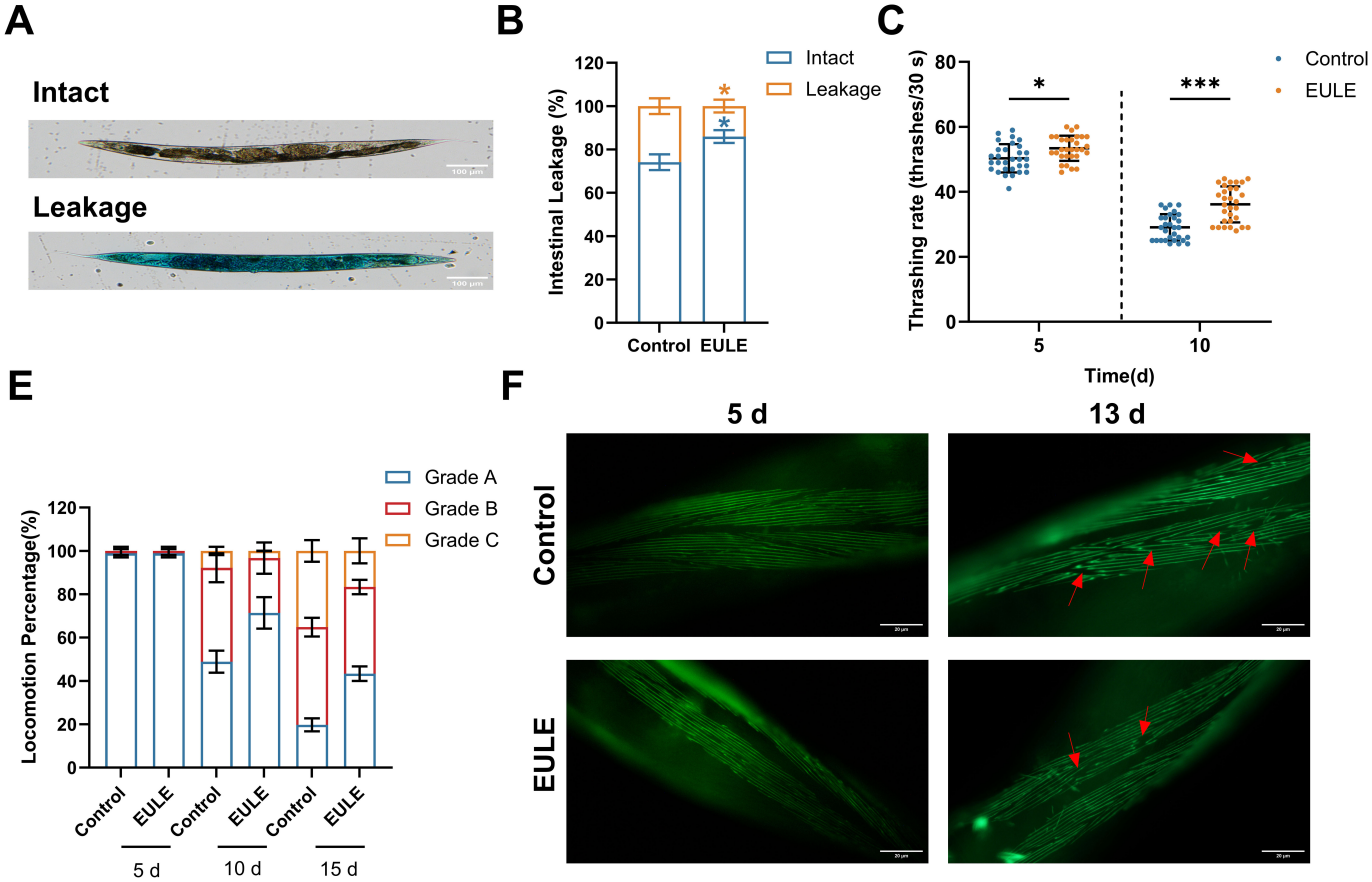
EULE ameliorates intestinal integrity and muscle function in *C. elegans*. **(A)** Representative images of wild-type N2 *C. elegans* after 15 days of EULE treatment, stained with food dye (brilliant blue), showing intact and leaky intestines. Scale bar: 100 μm. **(B)** Statistical analysis of intestinal integrity in wild-type N2 *C. elegans* after 15 days of EULE treatment, quantified by the number of intact vs. leaky intestines following food dye staining. **(C)** Thrashing frequency of wild-type N2 *C. elegans* within 30 s in M9 buffer after 5 and 10 days of EULE treatment. **(D)** Locomotor activity of wild-type N2 *C. elegans* after 5, 10, and 15 days of EULE treatment, assessed using a graded behavioral scoring system: Grade A: Normal movement. Grade B: Abnormal movement. Grade C: Only head or tail movement observed. **(E)** Representative images of myo-3p::GFP expression in transgenic *C. elegans* strain RW1596 after 5 and 13 days of EULE treatment. Scale bar: 20 μm. All statistical analyses were performed using unpaired two-tailed Student's *t-*test. *p < 0.05, ***p < 0.001.

The intestine of *C. elegans* serves as both a metabolic hub and a critical defense barrier, facilitating the exchange of water and nutrients while preventing the invasion of toxic substances. Previous studies have demonstrated that intestinal barrier function naturally declines during *C. elegans* aging, with significant dye leakage observed around day 15 of adulthood (44). In this study, intestinal integrity was assessed using food dye staining. Consistent with these reports, our results showed substantial intestinal barrier dysfunction in control worms at day 15. However, the proportion of *C. elegans* with intact intestines in the EULE treated group was significantly higher than that in the control group, showing a 16.04% increase (*p* < 0.05) (Figures 4A, B). These findings indicate that EULE effectively preserves intestinal barrier function in aging *C. elegan*s. The thrashing frequency of *C. elegans* in M9 buffer is a direct indicator of muscle system health. At day 5, the thrashing frequency in the EULE-treated group (53.40 ± 3.79 thrashes/30 s) was already significantly higher than that in the control group (50.33 ± 4.27 thrashes/30 s) (*p* < 0.05). The improvement became more pronounced at day 10, with the EULE group maintaining a higher thrashing frequency (36.13 ± 5.45 thrashes/30 s) compared to the control group (29.07 ± 3.98 thrashes/30 s) (*p* < 0.001) (Figure 4C).

The maintenance of muscle function was also reflected in locomotor behavior. Using a locomotion grading system, no significant difference was observed between the control and EULE groups at day 5 (*p* > 0.05). However, by day 10, the proportion of *C. elegans* retaining Grade A locomotion in the EULE treated group was significantly higher than that in the control group (EULE: 71.36 ± 5.92% vs. control: 48.89 ± 4.16%, *p* < 0.05). By day 15, the advantage of the EULE group became even more evident (Grade A proportion: EULE: 43.33 ± 2.72% vs. control: 19.75 ± 2.42%, *p* < 0.001; Grade C pro-portion: control: 16.67 ± 4.71% vs. EULE: 35.16 ± 4.19%, *p* < 0.05) (Figure 4D), suggesting that EULE enhances locomotor coordination during aging. Given the high homology between *C. elegans* muscle cells and those of mammals, aging in *C. elegans* is accompanied by disorganization and fragmentation of myofibers. Using the transgenic strain RW1596, we observed that by day 13, the control group exhibited typical aging phenotypes, including disorganized, wavy myofiber arrangements and compromised structural integrity. In contrast, the EULE-treated group largely maintained well-ordered parallel myofiber bundles with clear sarcomere structures and reduced myofiber breakage (Figure 4E).

### 3.7 EULE improves stress resistance and enhances the endogenous antioxidant system in *C. elegans*

**Figure 5:**
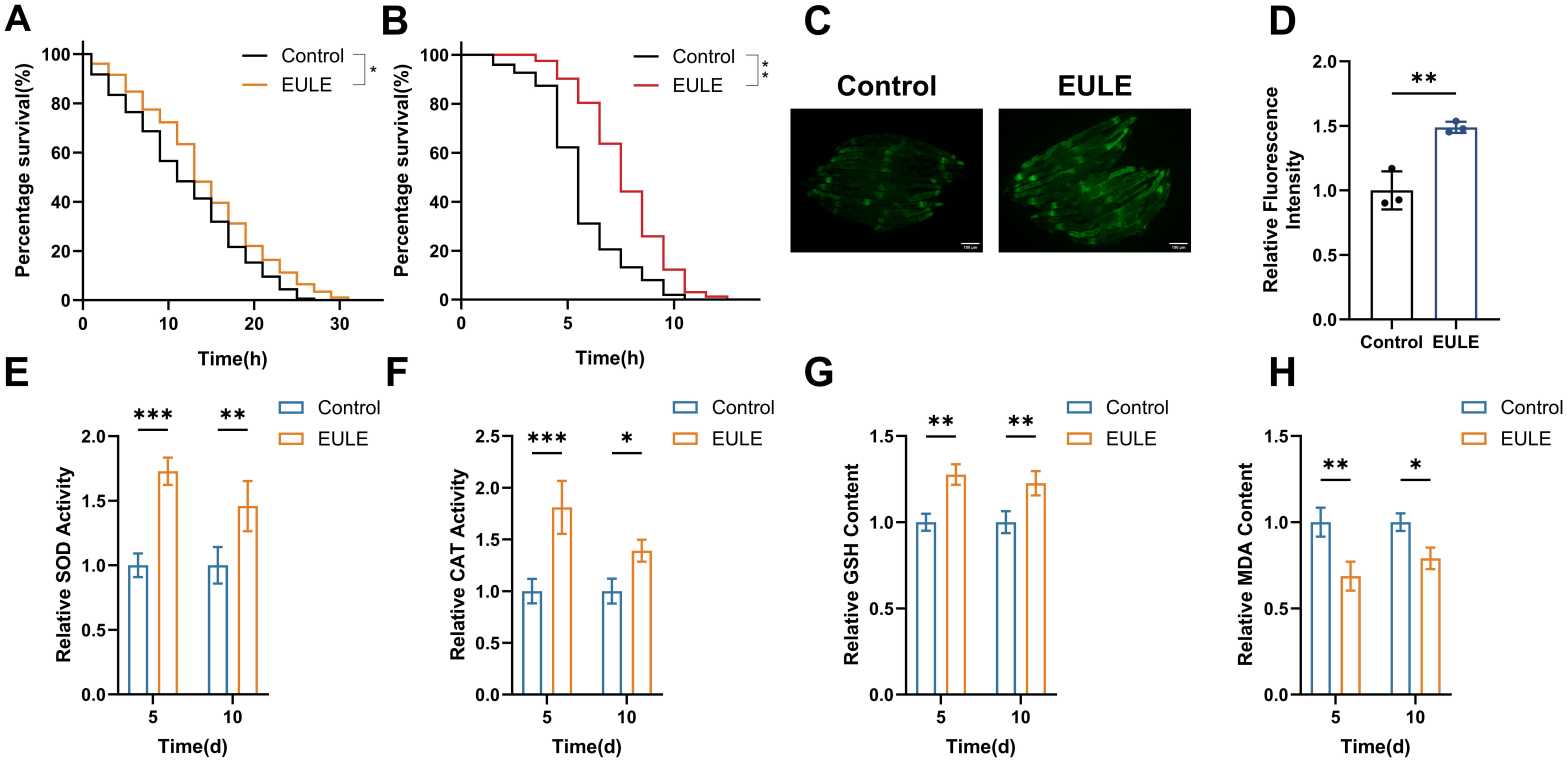
EULE augments the survival of *C. elegans* under oxidative and thermal stress conditions and enhances endogenous antioxidant system activity. **(A)** Survival analysis of wild-type N2 *C. elegans* exposed to EULU for 5 days under 300 μM juglone-induced oxidative stress. **(B)** Survival analysis of wild-type N2 *C. elegans* following 5-day EULU treatment under 35 °C heat stress. **(C)** Representative fluorescence micrographs depicting sod-3::GFP expression in transgenic CF1553 after 5 days of EULU administration. Scale bar: 100 μm. **(D)** Quantification of sod-3::GFP fluorescence intensity in transgenic CF1553 post 5-day EULU exposure. **(E–H)** Relative activity levels of SOD, CAT, GSH, and MDA in wild-type N2 *C. elegans* after 5 and 10 days of EULU treatment. Statistical significance was determined using unpaired two-tailed Student's *t-*test. *p < 0.05, **p < 0.01, ***p < 0.001.

Extensive research has established a positive association between organismal longevity and enhanced survival under oxidative and thermal stress. To determine whether EULE augments stress resilience in *C. elegans*, both juglone-induced oxidative stress and acute heat shock assays were performed. Juglone, a robust pro-oxidant, induces rapid intracellular accumulation of ROS, disrupting redox equilibrium and inflicting substantial oxidative injury, which consequently reduces lifespan in *C. elegans*. EULE administration significantly elevated survival rates under juglone-induced oxidative stress. The mean survival time in the EULE-treated group reached 15.31 ± 0.81 h, a 17.74% increase over the control group (13.01 ± 0.38 h; *p* < 0.01) (Figure 5A). Similarly, exposure to elevated temperatures (35 °C) precipitated cellular damage and accelerated senescence; however, EULE supplementation extended mean survival to 8.18 ± 0.27 h, representing a 14.79% improvement compared to controls (*p* < 0.01), thereby demonstrating enhanced thermotolerance (Figure 5B).

To elucidate the mechanistic basis of EULE's antioxidative effects, the transgenic strain CF1553 (sod-3::GFP) was utilized to assess SOD-3 expression, a pivotal antioxidant enzyme. EULE treatment markedly upregulated SOD-3, as evidenced by a 48.84% increase in fluorescence intensity relative to controls (*p* < 0.01) (Figures 5C, D). Enzymatic activity assays further revealed that SOD activity in the EULE group was significantly elevated by 72.81% (*p* < 0.001) and 45.84% (*p* < 0.01) on days 5 and 10, respectively, while CAT activity increased by 81.01% (*p* < 0.001) and 39.06% (*p* < 0.05). Additionally, non-enzymatic antioxidant GSH levels rose by 27.63% and 22.64% (*p* < 0.05) on days 5 and 10, respectively. This augmented antioxidant capacity effectively counteracted oxidative insult. Notably, EULE treatment significantly reduced MDA concentrations, a marker of lipid peroxidation, by 31.35% (*p* < 0.01) and 20.96% (*p* < 0.05) on days 5 and 10, respectively (Figures 5E–H). Collectively, these results indicate that EULE activates the antioxidant defense network, thereby enhancing stress resistance and mitigating oxidative damage in *C. elegans*.

### 3.8 EULE enhances mitochondrial function in *C. elegans*

**Figure 6:**
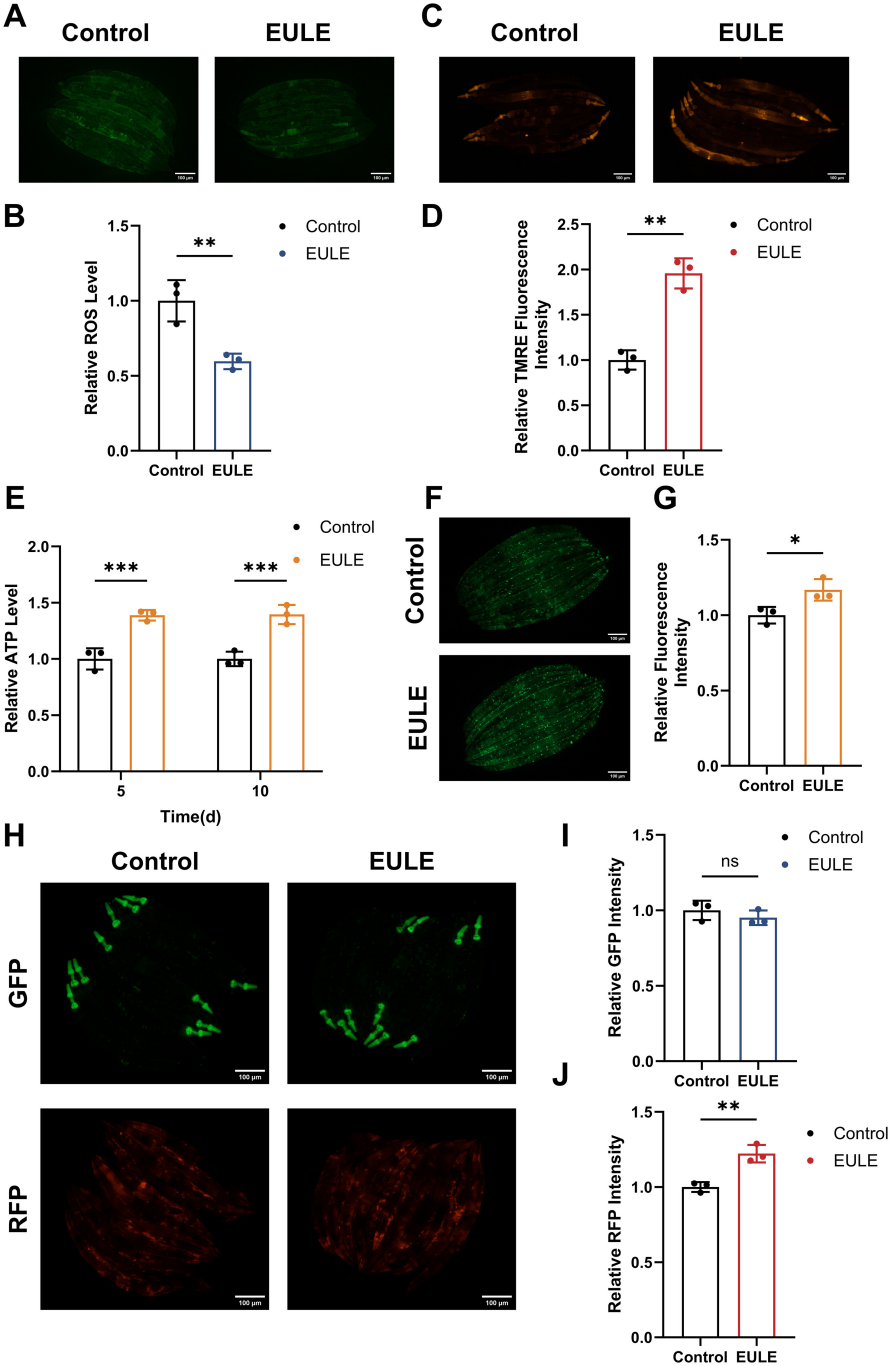
EULE attenuates ROS production, improves MMP, and maintains mitochondrial integrity in *C. elegans*. **(A)** Representative fluorescence micrographs depicting ROS levels in wild-type N2 *C. elegans* following 10 days of EULE administration, visualized with the H_2_DCF-DA probe. Scale bar: 100 μm. **(B)** Quantification of ROS-associated fluorescence intensity in wild-type N2 *C. elegans* after 10 days of EULE exposure, assessed via H_2_DCF-DA staining. **(C)** Representative images illustrating MMP in wild-type N2 *C. elegans*. **(D)** Quantitative analysis of MMP fluorescence intensity in wild-type N2 *C. elegans* post 10-day EULE treatment, determined using the TMRE probe. **(E)** Measurement of relative ATP concentrations in wild-type N2 *C. elegans* after 5 and 10 days of EULE intervention. **(F)** Representative fluorescence images of myo-3p::GFP expression in the transgenic PD4251 *C. elegans* strain following 10 days of EULE treatment. Scale bar: 100 μm. **(G)** Quantitative assessment of myo-3p::GFP fluorescence intensity in transgenic PD4251 *C. elegans* after 10 days of EULE exposure. **(H)** Representative fluorescence images of GFP and RFP in transgenic CB7272 *C. elegans* after 13 days of EULE treatment. Scale bar: 100 μm. **(I)** Quantification of GFP fluorescence intensity in transgenic CB7272 *C. elegans* after 13 days of EULE administration. **(J)** Quantification of RFP fluorescence intensity in transgenic CB7272 *C. elegans* after 13 days of EULE administration. All statistical analyses were conducted using unpaired two-tailed Student's *t-*test. *p < 0.05, **p < 0.01, ***p < 0.001.

Previous research has established that EULE markedly augments antioxidant enzyme activity in *C. elegans*. The onset and progression of aging are governed by intricate molecular pathways, intimately linked to the pathological accumulation of ROS. When ROS generation surpasses the neutralizing capacity of endogenous antioxidant defenses, oxidative stress ensues, resulting in bio-macromolecular damage and expedited senescence. To elucidate whether EULE modulates this process, intracellular ROS levels were quantified in *C. elegans* utilizing the H_2_DCF-DA fluorescent probe. EULE administration led to a 40.41% reduction in ROS levels relative to controls (*p* < 0.01) (Figures 6A, B), signifying a robust attenuation of oxidative stress.

Given the centrality of mitochondria as both the predominant source (~90%) and principal target of intracellular ROS, oxidative stress is intrinsically associated with mitochondrial dysfunction. We postulated that the antioxidative efficacy of EULE may be attributable to its capacity to preserve mitochondrial integrity. MMP, a key biomarker of mitochondrial health and electron transport chain (ETC) competency, was assessed using the TMRE fluorescent probe. EULE treatment elicited a 48.88% elevation in TMRE fluorescence intensity compared to controls (*p* < 0.01) (Figures 6C, D), indicating a significant enhancement of MMP and, by extension, mitochondrial function.

Maintenance of optimal MMP is essential for efficient ATP biosynthesis, a critical determinant of longevity. Accordingly, we evaluated the impact of EULE on ATP concentrations in *C. elegans*. EULE supplementation resulted in a significant increase in ATP content in both young and aged worms, with increments of 27.38% and 26.09% at days 5 and 10, respectively (*p* < 0.001) (Figure 6E). Furthermore, mitochondrial abundance was assessed using the PD4251 transgenic strain, which expresses mitochondria-targeted GFP in body wall muscle cells. EULE treatment yielded a 16.84% increase in GFP fluorescence intensity (*p* < 0.05) compared to controls (Figures 6F, G), reflecting augmented mitochondrial biogenesis. Collectively, these findings demonstrate that EULE mitigates aging by enhancing mitochondrial function, elevating MMP, and promoting mitochondrial biogenesis.

### 3.9 EULE protects mitochondrial function in *C. elegans* by upregulating respiratory chain complexes IV and V

Mitochondria, often referred to as the cellular “powerhouses,” are indispensable for sustaining cellular bioenergetics and mitigating senescence. The respiratory chain complexes I–V, localized within the mitochondrial inner membrane, form the central machinery of the oxidative phosphorylation (OXPHOS) system, mediating electron transfer and establishing electrochemical proton gradients essential for ATP biosynthesis. The structural fidelity and enzymatic competence of these complexes are pivotal for optimal mitochondrial energy transduction. Importantly, dysfunction, aberrant assembly, or diminished activity of respiratory chain complexes—particularly complexes I, III, and IV—are well-established molecular signatures and mechanistic drivers of mitochondrial aging.

In transgenic CB7272 *C. elegans*, GFP selectively tags subunits of complexes I, II, and III in the pharyngeal and body wall musculature, while RFP labels complexes IV (cytochrome c oxidase) and V (ATP synthase) in the pharynx and epidermis. The impact of EULE on mitochondrial respiratory chain integrity was assessed by quantifying GFP and RFP fluorescence. Relative to controls, EULE treatment did not significantly alter GFP fluorescence intensity (*p* > 0.05), whereas RFP fluorescence exhibited a 22.21% increase (*p* < 0.01) (Figures 6H–J). These findings indicate that EULE may confer mitochondrial protection by upregulating or stabilizing the terminal respiratory chain complexes IV and V, which are integral to terminal electron transfer and ATP generation. Given the central roles of complexes IV and V in OXPHOS and cellular energy equilibrium, their augmented expression may directly enhance mitochondrial performance, boost ATP output, and counteract age-related declines in bioenergetic capacity, thereby promoting mitochondrial health.

### 3.10 Transcriptomic analysis of EULE-treated in *C. elegans*

**Figure 7:**
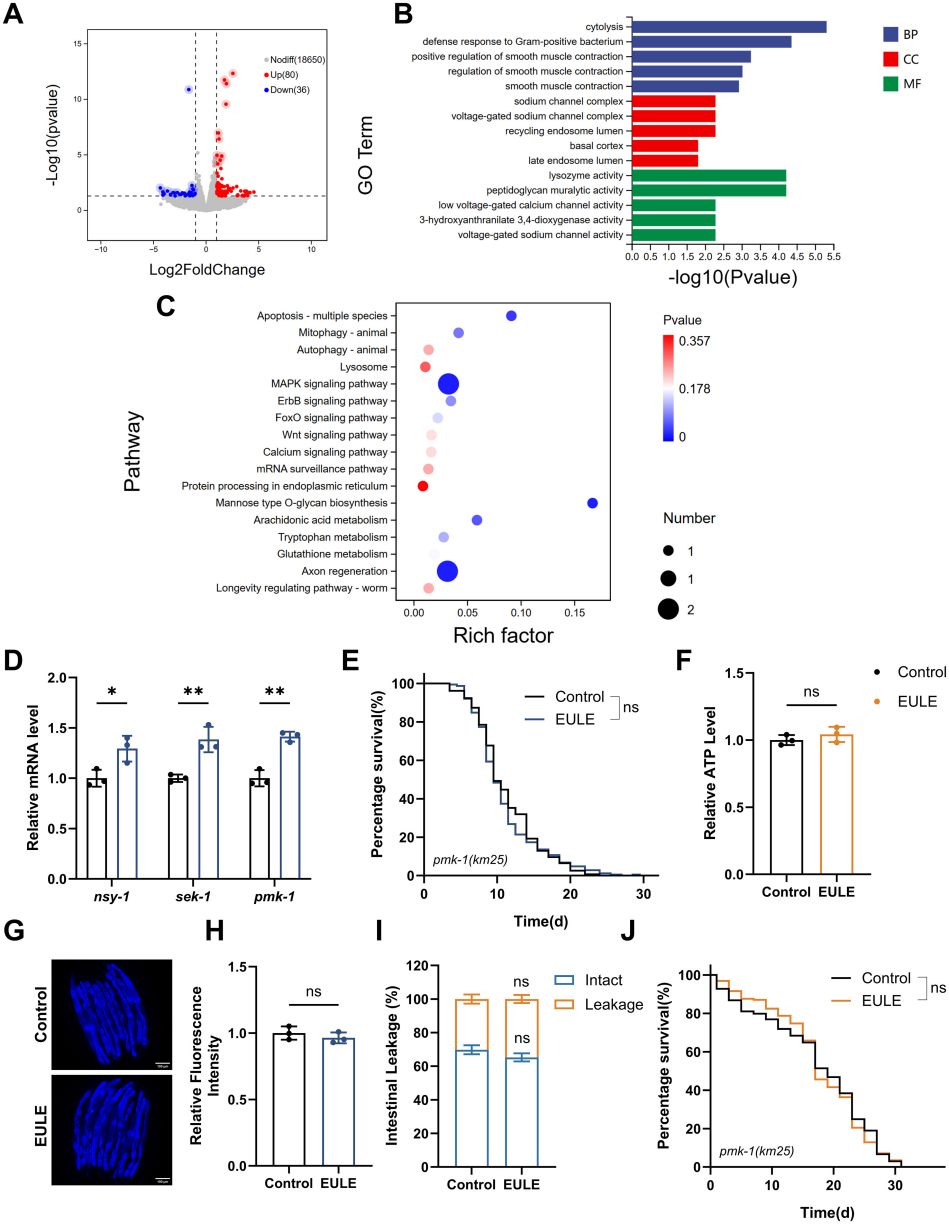
EULE-induced lifespan extension in *C. elegans* is linked to the MAPK signaling pathway. **(A)** Volcano plot illustrating differentially expressed genes between control and EULE-treated groups. **(B)** Top 20 significantly enriched metabolic pathways identified via GO analysis. **(C)** Top 20 significantly enriched metabolic pathways identified through KEGG pathway analysis. **(D)** Quantitative expression profiles of *nsy-1, sek-1*, and *pmk-1* following five days of EULE administration. **(E)** Kaplan-Meier survival analysis of *pmk-1* (*km25*) mutants subjected to EULE treatment. **(F)** Relative ATP quantification in *pmk-1* (*km25*) mutants post-EULE exposure. **(G)** Representative micrographs depicting lipofuscin accumulation in *pmk-1* (*km25*) mutants after EULE intervention; scale bar: 100 μm. **(H)** Quantitative assessment of lipofuscin levels in *pmk-1* (*km25*) mutants following EULE treatment. **(I)** Statistical evaluation of intestinal barrier integrity (intact vs. compromised) in *pmk-1* (*km25*) mutants after 15 days of EULE exposure, determined by food dye permeability assay. **(J)** Kaplan-Meier survival curves of *pmk-1* (*km25*) mutants under juglone-induced oxidative stress subsequent to EULE treatment. All statistical analyses were conducted using unpaired two-tailed Student's *t-*test. *p < 0.05, **p < 0.01.

To investigate the molecular pathways through which EULE prolongs the lifespan of *C. elegans*, we conducted transcriptomic profiling via RNA sequencing. Relative to the control group, EULE administration induced significant alterations in the gene expression landscape, resulting in the identification of 116 differentially expressed genes (DEGs), comprising 80 upregulated and 36 downregulated transcripts (*p* < 0.05, |log2FC| > 1) (Figure 7A). GO enrichment analysis indicated that EULE primarily exerts its effects by enhancing the innate immune response and defense mechanisms in *C. elegans*. Biological process (BP) analysis demonstrated that the DEGs were significantly enriched in “defense response to other organisms,” “response to biotic stimulus,” and related cross-species interaction processes. Molecular function (MF) analysis indicated that the most significantly enriched terms were “lysozyme activity” and “peptidoglycan muralytic activity,” both of which serve as direct mechanisms against bacterial invasion. Meanwhile, cellular component (CC) analysis showed that these defense-related proteins were primarily localized in the “extracellular region,” suggesting their secretion for extracellular defense functions. Additionally, the enrichment of “lysosome” and “endopeptidase activity” indicated enhanced intracellular clearance of pathogens and damaged macromolecules (Figure 7B).

KEGG pathway enrichment analysis clearly highlighted the “MAPK signaling pathway,” a central hub in *C. elegans* that integrates stress signals and regulates innate immunity and longevity. These results indicate that EULE may promote organismal homeostasis and healthspan by activating adaptive defense responses via the MAPK pathway. The activation of the “MAPK signaling pathway,” in conjunction with the “Apoptosis” pathway, facilitates the removal of damaged or dysfunctional cells, serving as a key mechanism of tissue quality control. In addition, enrichment of the “Mannose-type O-glycan biosynthesis” pathway implies enhanced protein stability and function through glycosylation modifications, thereby contributing to improved proteostasis. Notably, the significant enrichment of the “Axon regeneration” pathway suggests that EULE may confer neuroprotective benefits by promoting neuronal repair and maintenance during aging (Figure 7C).

Taken together, these findings suggest that EULE exerts its effects not through a singular molecular target, but by engaging a complex, multi-tiered protective network predominantly organized around the MAPK signaling pathway. This network coordinates innate immune responses and additional defense pathways, working in concert to decelerate the aging process in *C. elegans*.

### 3.11 EULE extends the lifespan of *C. elegans* through activation of the MAPK signaling pathway

To further dissect the role of the MAPK pathway in EULE-mediated longevity in *C. elegans*, we conducted additional analysis of our transcriptomic data. The results revealed significant downregulation of core components of the JNK signaling pathway (*kgb-2* and *cca-1*), suggesting that the lifespan-extending effect of EULE is unlikely to be mediated through the JNK pathway. We therefore shifted our focus to the *pmk-1*/p38 MAPK pathway, a branch critically involved in stress response and innate immunity. Although the transcriptional level of *pmk-1* itself remained unchanged—consistent with its known activation via post-translational phosphorylation—we observed a marked and systematic upregulation of its well-established downstream target genes. These included canonical innate immune effectors such as lysozymes (*ilys-2, ilys-3*, and *ilys-5*) and C-type lectins (*clec-80, clec-218*, and *clec-263*) (Supplementary Data S1). This coordinated upregulation serves as a robust molecular signature of *pmk-1* pathway activation. In *C. elegans*, the MAPK signaling pathway, comprising *nsy-1* (a MAP Kinase Kinase Kinase, or MAPKKK), *sek-1* (a MAP Kinase Kinase, or MAPKK), and *pmk-1* (the p38 MAPK ortholog), functions as a conserved regulatory axis modulating innate immunity, stress resilience, and organismal longevity. To elucidate whether EULE-mediated lifespan extension operates via MAPK pathway activation, RT-qPCR analysis was conducted to quantify the transcript levels of pivotal MAPK components in wild-type N2 *C. elegans* post-EULE exposure. EULE treatment elicited a significant upregulation of *nsy-1, sek-1*, and *pmk-1* mRNA expression (*p* < 0.01 for all; Figure 7D), signifying robust pathway activation. To substantiate the requirement of this signaling module, a *pmk-1* loss-of-function mutant was examined. Notably, EULE failed to prolong lifespan in the *pmk-1* mutant, as no statistical difference was observed between EULE-treated and control groups (mean lifespan: 11.54 ± 0.31 d vs. 11.85 ± 0.93 d, *p* > 0.05; Figure 7E). Additionally, EULE did not confer improvements in healthspan metrics, including ATP content, lipofuscin clearance, intestinal barrier preservation, or oxidative stress resistance in the *pmk-1*-deficient background (Figures 7F–J). Collectively, these results establish the indispensability of the MAPK pathway for EULE-induced longevity and healthspan benefits, with mechanistic evidence indicating that EULE augments *nsy-1* and *sek-1* transcription and necessitates intact *pmk-1* function to orchestrate systemic aging delay in *C. elegans*.

## 4 Discussion

Owing to progress in biomedical research and enhancements in public health infrastructure, worldwide life expectancy has risen markedly over recent decades. Nevertheless, gains in healthy life expectancy have lagged behind, primarily as a result of the growing prevalence of chronic non-communicable diseases. This trend has contributed to a deterioration in the overall quality of life among older populations (45). Consequently, promoting healthy aging and enhancing health status in later life have emerged as core strategies to address the challenges posed by population aging. Aging is fundamentally a progressive decline in organ function and accumulation of pathological damage across multiple systems, necessitating long-term, multidimensional interventions for effective mitigation (2). TCM, guided by holistic principles, syndrome differentiation, and preventive treatment concepts, has accumulated unique theoretical foundations and clinical experience in chronic disease management and functional maintenance, demonstrating irreplaceable advantages and broad application prospects (46). Consequently, the systematic screening and identification of safe and efficacious anti-aging bioactive compounds from natural sources, especially traditional herbs with well-documented therapeutic use, is of substantial practical importance. Herbs classified as both medicinal and edible have attracted significant interest in health-related interventions due to their established safety profiles and suitability for consumption (47). EUL can be seamlessly integrated into routine nutrition via functional teas, nutraceutical supplements, or as food additives, thereby enhancing compliance and practicality in health intervention protocols. This underscores EUL's potential as a pivotal agent in key public health sectors, such as chronic disease prevention, suboptimal health state regulation, and the advancement of healthy aging initiatives (48–50).

To elucidate the material basis underlying the anti-aging activity of EULE, this study systematically identified its chemical constituents using UPLC-Q-Exactive Orbitrap MS. The results revealed a rich profile of bioactive compounds, with iridoids (e.g., geniposidic acid, asperuloside), phenylpropanoids (e.g., chlorogenic acid), flavonoids (e.g., quercetin, kaempferol), and lignans being representative active components (Figure 1). These compounds provide crucial clues for understanding EULE's mechanism of action, particularly their collective ability to modulate the p38/MAPK signaling pathway. Extensive evidence indicates that the p38 MAPK pathway is a primary convergent target for these diverse chemical classes (51, 52). For instance, iridoids like geniposidic acid and asperuloside, phenylpropanoids like chlorogenic acid, and lignans have all been documented to inhibit p38 MAPK phosphorylation in various mammalian models of inflammation and stress (53–55). It is crucial to note that these studies often report inhibition in the context of pathological hyper-activation (e.g., by LPS). In the context of aging in *C. elegans*, the *pmk-1*/p38 pathway primarily governs innate immunity and stress resistance, where its controlled activation is beneficial for longevity (56, 57). Therefore, we hypothesize that the bioactive compounds in EULE do not act as simple inhibitors but as sophisticated modulators. They may induce a mild, salutary stress that leads to a beneficial, adaptive activation of the *pmk-1* pathway, or they may fine-tune the pathway's sensitivity to endogenous age-related stress. The dual role of flavonoids like quercetin, which can either inhibit or activate p38 MAPK depending on the cellular context, exemplifies this complexity. In summary, the broad-spectrum anti-aging efficacy of EULE is not attributed to a single constituent but rather results from a synergistic network. This “poly-pharmacology” orchestrated by its iridoids, flavonoids, and lignans converges on the modulation of the central *pmk-1*/p38 MAPK signaling hub, thereby enhancing stress resilience, maintaining muscular and intestinal integrity, and ultimately extending healthspan.

Aging represents a complex biological phenomenon marked by progressive functional deterioration across multiple physiological systems in *C. elegans* (6). In this investigation, EULE administration not only significantly prolonged the mean lifespan of wild-type N2 *C. elegans* but, more notably, enhanced healthspan by preserving pharyngeal pumping rates and locomotor function in senescent worms (Figures 3A, B), while substantially inhibiting the accumulation of the aging biomarker lipofuscin (Figures 3E, F). Recognizing that stress resilience is a pivotal factor influencing longevity (58), we comprehensively assessed the modulatory impact of EULE on stress tolerance in *C. elegans*. Our findings demonstrated that EULE treatment markedly improved survival under heat stress and conferred greater resistance to juglone-induced acute oxidative insult (Figures 5A, B). Further analysis of key redox homeostasis parameters indicated that EULE robustly activated the endogenous antioxidant defense machinery, as reflected by increased activities of SOD and CAT. This upregulation of antioxidant enzymes facilitated efficient clearance of intracellular ROS, thereby attenuating oxidative stress-mediated lipid peroxidation and significantly lowering MDA concentrations (Figures 5C–H).

Age-associated sarcopenia and compromised intestinal barrier function are recognized as pivotal factors in the physiological decline observed during aging (59, 60). In our study, EULE administration significantly attenuated sarcopenic progression in *C. elegans*, preserving the organized architecture of muscle fibers in aged, treated specimens and mitigating degenerative morphological disruptions. This maintenance of muscular structural integrity underpins the sustained locomotor performance observed in elderly worms (Figure 4E). Furthermore, EULE demonstrated a robust capacity to preserve intestinal barrier function, a determinant of immunological and metabolic equilibrium. Intestinal permeability assays revealed that EULE substantially reduced age-related intestinal leakage in senescent worms. Collectively, these results indicate that EULE fortifies intestinal barrier integrity, thereby restricting the translocation of deleterious luminal agents—a fundamental mechanism for alleviating systemic aging stress and supporting physiological homeostasis (Figures 4A, B).

Mitochondrial dysfunction, a recognized hallmark of aging, promotes cellular senescence by impairing bioenergetic capacity and disrupting redox homeostasis (1, 61). Our findings suggest that mitochondria represent critical organelle targets mediating the anti-aging properties of EULE. Administration of EULE not only attenuated intracellular ROS accumulation but also markedly enhanced the expression of essential mitochondrial respiratory chain components, specifically Complex IV (cytochrome c oxidase) and Complex V (ATP synthase) (Figure 6). This upregulation facilitated improved ETC performance and coupling efficiency, thereby stabilizing MMP. Given that MMP underpins the electrochemical gradient necessary for proton translocation and ATP generation, its maintenance is vital for optimal oxidative phosphorylation (62, 63). Restoration of MMP was paralleled by a recovery of ATP synthesis in EULE-treated worms, indicating that EULE mitigates age-associated declines in cellular bioenergetics and supports organismal physiological integrity. Transcriptomic analysis further untangled the underlying molecular mechanisms by which EULE preserves mitochondrial function, primarily through the systematic regulation of multiple genes associated with mitochondrial activity. Specifically, EULE not only upregulated gpx-3, which is responsible for ROS clearance, and mct-2, which facilitates energy substrate transport, but also potentially modulated ncx-9, a key gene involved in mitochondrial calcium homeostasis, thereby collectively maintaining the stability of the mitochondrial internal environment. This multi-target regulatory pattern highlights the comprehensive role of EULE in restoring mitochondrial health. Crucially, this restoration of mitochondrial integrity is not merely a passive recovery of energy production; it functions as a critical upstream event that generates intracellular signals to orchestrate cellular stress responses and longevity pathways.

A pivotal question arising from our findings is how the observed improvements in mitochondrial function are translated into a systemic, pro-longevity program. The link is not coincidental but mechanistic, rooted in the well-established “mitochondria-p38 MAPK signaling axis” (64, 65). Mitochondria serve as primary sensors of cellular stress, and subtle shifts in their function, particularly the generation of reactive oxygen species (ROS), act as potent upstream signals that activate the MAPK cascade (66). Specifically, mitochondrial ROS can trigger the activation of upstream kinases like *ask1*/*nsy-1*, which in turn phosphorylates and activates the *sek-1* and subsequently the *pmk-1*/p38 pathway (67–69). Therefore, we propose a clear mechanistic model: the bioactive compounds in EULE first modulate mitochondrial function, leading to an adaptive signaling output (e.g., a controlled level of ROS). This mitochondrial signal is then sensed and transduced through the *nsy-1, sek-1*, and *pmk-1* cascade. In this network, *pmk-1* serves as the central integrator, translating the upstream mitochondrial status report into a coordinated organismal stress response and longevity program. This model explains why improvements in mitochondrial bioenergetics are directly coupled to the activation of a key stress-response pathway. Furthermore, this signaling is often bidirectional; activated p38 MAPK can in turn regulate mitochondrial biogenesis and quality control, forming a feedback loop that reinforces cellular homeostasis and resilience against age-related decline (56, 70). Targeted validation in our study confirmed that genetic knockout of *pmk-1* completely abrogated EULE-induced longevity and its associated benefits, establishing the *pmk-1*/p38 MAPK pathway as the essential downstream effector axis that translates mitochondrial functional improvements into extended lifespan.

In this study, our experimental validation focused on the MAPK signaling pathway, which exhibited the most significant enrichment in the RNA-seq data. However, the transcriptomic data also revealed significant enrichment in other pathways closely associated with the aging process, such as “Apoptosis” “Axon regeneration” and “Mannose type O-glycan biosynthesis” (Figure 7). These pathways play pivotal roles in clearing senescent cells to maintain tissue homeostasis, supporting neural plasticity, and regulating key protein modifications, respectively. Collectively, these findings suggest that the anti-aging efficacy of EUL involves a complex regulatory network of multiple pathways and targets. To maintain the focus and depth of this investigation, we did not experimentally validate each of these pathways individually. Nonetheless, these data highlight the broad spectrum of the anti-aging potential of EUL and open new avenues for future research. For instance, future studies of great value could investigate whether EUL modulates apoptosis to precisely clear senescent cells, or whether its promoting effect on axon regeneration translates into functional neural improvements.

We should also acknowledge the limitations of our study. The transcriptomic analysis was conducted at a single time point, day 5 of adulthood. While this strategy was optimal for identifying the early, upstream regulatory pathways modulated by EULE, we recognize that aging is a dynamic process. The molecular effects of EULE may evolve throughout the organism's lifespan. Therefore, future time-course studies, for instance, analyzing the transcriptome at later stages such as day 10 or day 15, would be highly valuable.

In conclusion, this investigation reveals that EULE prolongs lifespan and mitigates age-associated phenotypes in *C. elegans* by first enhancing mitochondrial bioenergetics and maintaining mitochondrial homeostasis, which in turn activates the *pmk-1*/p38 MAPK signaling pathway to orchestrate a robust antioxidant and stress response program. These results furnish empirical support for the geroprotective potential of EUL and lay a conceptual groundwork for their integration into functional food formulations and pharmaceutical development. Nevertheless, due to pronounced physiological disparities between *C. elegans* and mammalian systems, it is crucial to emphasize that the effective concentrations identified in our study cannot be directly extrapolated to establish a safe or effective dose for human consumption. Therefore, further studies employing higher-order animal models are necessary to elucidate the bioactive components, delineate their mechanistic interactions, and assess the evolutionary conservation of EULE's anti-aging efficacy. This cautionary approach is essential to prevent the potential misuse of traditional medicines based on preclinical data and to ensure a safe transition toward any potential clinical application.

## 5 Conclusions

The results of this investigation indicate that EULE may promote both lifespan extension and improved healthspan in *C. elegans* by modulating the p38 MAPK signaling pathway. The array of phytochemicals present in EULE likely serve as exogenous modulators, triggering activation of the p38 MAPK pathway, which plays a pivotal role in innate immune responses and cellular stress adaptation. This pathway activation orchestrates the upregulation of endogenous antioxidant defense mechanisms, sustains mitochondrial functional homeostasis, and preserves tissue structural integrity, collectively enhancing the organism's homeostatic balance and adaptability to environmental challenges. These findings offer molecular-level experimental validation of EUL's bioactivity and imply that its physiological effects are mediated via a synergistic, multi-component, and multi-target mechanism. In conclusion, this study provides a robust scientific rationale for the application of EUL in the development of functional foods and dietary supplements aimed at enhancing healthspan.

## Data Availability

The original contributions presented in the study are publicly available. This data can be found in the NCBI repository, accession number: PRJNA1345583.
