# Supplementary material for: Phytochemical-rich Eucommia ulmoides leaf extract extends healthspan in Caenorhabditis elegans via the pmk-1/p38 MAPK pathway and mitochondrial homeostasis
Source: Front Nutr. 2025 Oct 27;12:1680518. doi: 10.3389/fnut.2025.1680518 (PMC12597746; doi:10.3389/fnut.2025.1680518)
Supplement: Supplementary file 1 [file Table_1.docx]

Supplementary Material

**Table S1** **Primer sequences used for real-time PCR.**

| Gene name | Primer sequences (5'-3') |
| --- | --- |
| *nsy-1* | F: ACAAGAGGCAAGTGCAGCAT |
|  | R: TGTCAAAATCGATGCGCGTT |
| *sek-1* | F: TGGAAGTGATGGACACGTCG |
|  | R: TCCGGCTTGTACAGTCTTGG |
| *pmk-1* | F: ATGGAACTGTTTGTGCTGCTG |
|  | R: TCACGATATGTACGACGGGC |
| *act-1* | F: GGCCCAATCCAAGAGAGGTAT |
|  | R: GGGCAACACGAAGCTCATTG |

**Table S2 Qualitative analysis of chemical constituents from the EULE.**

| **No.** | | **RT**  **（min）** | **Components** | | | **Formula** | | **Ion Mode** | | **Measured**  **m/z** | | **Calculated**  **m/z** | | **Mass Error**  **/ppm** | | **MS/MS** | **Class** | |
| --- | --- | --- | --- | --- | --- | --- | --- | --- | --- | --- | --- | --- | --- | --- | --- | --- | --- | --- |
| 1 | | 0.93 | Gluconic acid | | | C_6_H_12_O_7_ | | [M-H]^-^ | | 195.0501 | | 195.051 | | -4.61 | | 177.0401 | Organic acids | |
| 2 | | 0.94 | Quinic acid | | | C_7_H_12_O_6_ | | [M-H]^-^ | | 191.0561 | | 191.0552 | | 4.71 | | 129.0181,111.0074 | Organic acids | |
| 3 | | 1.17 | Citric acid | | | C_6_H_8_O_7_ | | [M-H]^-^ | | 191.0189 | | 191.0197 | | -4.19 | | 173.0081,129.0180,111.0074,87.0073,85.0073 | Organic acids | |
| 4 | | 1.44 | Deacetylasperulosidic acid* | | | C_16_H_22_O_11_ | | [M-H]^-^ | | 389.1096 | | 389.1089 | | 1.8 | | 227.0554,209.0447,183.0653,165.0541,147,0438 | Iridoids | |
| 5 | | 1.78 | DL-Malic acid | | | C_4_H_6_O_5_ | | [M-H]^-^ | | 133.0131 | | 133.0142 | | -8.27 | | 115.0023,89.0229,71.0123 | Organic acids | |
| 6 | | 1.98 | Protocatechuicacid-4-O-beta-glucoside | | | C_13_H_16_O_9_ | | [M-H]^-^ | | 315.0721 | | 315.0722 | | -0.32 | | 153.0182,108.0204 | Phenolic acids | |
| 7 | | 2.01 | 3-hydroxy-3-methylglutaric acid | | | C_6_H_10_O_5_ | | [M-H]^-^ | | 161.0444 | | 161.0455 | | -6.83 | | 143.0341,99.0438 | Organic acids | |
| 8 | | 2.04 | Glucosyringic acid | | | C_15_H_20_O_10_ | | [M-H]^-^ | | 359.0992 | | 359.0983 | | 2.51 | | 197.0447 | Phenolic acids | |
| 9 | | 2.31 | Pantothenic acid | | | C_9_H_17N_O_5_ | | [M+H]^+^ | | 220.118 | | 220.1179 | | 0.45 | | 124.0759,98.0242,90.0555,85.0654,72.0451 | Others | |
| 10 | | 2.45 | Geniposidic acid* | | | C_16_H_22_O_10_ | | [M^-^H]^-^ | | 373.114 | | 373.114 | | 0 | | 211.0605,193.0498,167.0703,149.0596,123.0439 | Iridoids | |
| 11 | | 2.5 | Protocatechuic acid | | | C_7_H_6_O_4_ | | [M-H]^-^ | | 153.0183 | | 153.0193 | | -6.54 | | 109.0281 | Phenolic acids | |
| 12 | | 3.04 | 3-Methoxybenzaldehyde | | | C_8_H_8_O_2_ | | [M+H]^+^ | | 137.0598 | | 137.0597 | | 0.73 | | 122.0365,94.0418 | Others | |
| 13 | | 3.21 | Chlorogenic acid* | | | C_16_H_18_O_9_ | | [M-H]^-^ | | 353.0877 | | 353.0878 | | -0.28 | | 191.055,3,179.0340，173.0444,135.0439 | Phenylpropanoids | |
| 14 | | 3.73 | Strophanthobiose | | | C_13_H_24_O_9_ | | [M+H]^-^ | | 323.1348 | | 323.1348 | | 0 | | 179.0556,161.0445 | Others | |
| 15 | | 3.74 | Protocatechuicaldehyde | | | C_7_H_6_O_3_ | | [M+H]^+^ | | 139.0391 | | 139.0389 | | 1.44 | | 121.0649,111.0444,93.0340,65.0394 | Phenylpropanoids | |
| 16 | 4.12 | | | 3,4-Dimethylbenzoicacid | C_9_H_10_O_2_ | | [M+H]^+^ | | 151.0755 | | 151.0753 | | 1.32 | | 121.0649,105.0703,91.0548,79.0549 | | | Organic acids |
| 17 | 4.46 | | | Catechin* | C_15_H_14_O_6_ | | [M+H]^+^ | | 291.0862 | | 291.0863 | | -0.34 | | 179.0701,123.0443 | | | Flavonoids |
| 18 | 4.56 | | | Asperulosidic acid* | C_18_H_24_O_12_ | | [M-H]^-^ | | 431.1192 | | 431.1194 | | -0.46 | | 251.0558,165.0545,119.0339,89.0230,71.0123,59.0124 | | | Iridoids |
| 19 | 4.68 | | | Mandelicacid | C_18_H_24_O_12_ | | [M-H]^-^ | | 431.1188 | | 431.1194 | | -1.39 | | 269.0672,225.0763,207.0652 | | | Organic acids |
| 20 | 4.77 | | | Cryptochlorogenic acid | C_16_H_18_O_9_ | | [M+Na]^+^ | | 377.0845 | | 377.0843 | | 0.53 | | 359.0746,331.0728,215.0527,197.0427,185.0215 | | | Phenylpropanoids |
| 21 | 5.03 | | | Aesculetin | C_9_H_6_O_4_ | | [M+H]^+^ | | 179.0339 | | 179.0338 | | 0.56 | | 133.0285,123.0443 | | | Coumarins |
| 22 | 5.9 | | | 7-Hydroxycoumarin | C_9_H_6_O_3_ | | [M+H]^+^ | | 163.039 | | 163.0389 | | 0.61 | | 145.0286,135.0442,117.0702,107.0859,89.0392 | | | Coumarins |

**Table S2. Cont.**

| **No.** | **RT**  **（min）** | **Components** | **Formula** | **Ion Mode** | **Measured**  **m/z** | **Calculated**  **m/z** | **Mass Error**  **/ppm** | **MS/MS** | **Class** |
| --- | --- | --- | --- | --- | --- | --- | --- | --- | --- |
| 23 | 6.09 | Asperuloside* | C_18_H_22_O_11_ | [M-H]^-^ | 413.1093 | 413.1089 | 0.97 | 191.0346,147.0440 | Iridoids |
| 24 | 6.21 | 4-Coumaric acid | C_9_H_8_O_3_ | [M+H]^+^ | 165.0549 | 165.0546 | 1.82 | 147.0442,119.0495 | Phenylpropanoids |
| 25 | 6.96 | Epicatechin | C_15_H_14_O_6_ | [M+H]^+^ | 291.0861 | 291.0863 | -0.69 | 207.0652,179.0704,165.0547,147.0441,139.0390,123.0443 | Flavonoids |
| 26 | 7.45 | 4-p-Coumaroylquinic acid | C_16_H_18_O_8_ | [M-H]^-^ | 337.0927 | 337.0928 | -0.3 | 191.0554,173.0446,163.0391,93.0331 | Phenolic acids |
| 27 | 7.66 | Olivil4'-O-glucoside | C_26_H_34_O_12_ | [M+HCOO]^-^ | 583.2043 | 583.2032 | 1.89 | 537.2623,375.1449,327.1241 | Lignans |
| 28 | 7.79 | B2Riboflavin | C_17_H_20N4_O_6_ | [M+H]^+^ | 377.1457 | 377.1455 | 0.53 | 243.0878 | Others |
| 29 | 7.98 | Luteolin-7,3'-di-O-glucoside | C_27_H_30_O_16_ | [M^-^H]^-^ | 609.1464 | 609.1461 | 0.49 | 447.0932,285.0406 | Flavonoids |
| 30 | 8.83 | Ferulic acid | C_10_H_10_O_4_ | [M+H-H_2_O]^+^ | 177.0548 | 177.0546 | 1.13 | 149.0600,117.0338,91.0548,89.0392 | Phenolic acids |
| 31 | 9.12 | 4-O-Feruloylquinicacid | C_17_H_20_O_9_ | [M-H]^-^ | 367.1035 | 367.1034 | 0.27 | 191.0553,93.0332 | Organic acids |
| 32 | 10.26 | Quercetin3,4'-diglucoside | C_27_H_30_O_17_ | [M-H]^-^ | 625.14 | 625.141 | -1.6 | 463.0887,301.0352 | Flavonoids |
| 33 | 10.29 | Myricetin3-O-beta-D-galactopyranoside | C_21_H_20_O_13_ | [M-H]^-^ | 479.0834 | 479.0831 | 0.63 | 316.0224 | Flavonoids |
| 34 | 11.06 | Quercetin3-(2R-apiosylrutinoside) | C_32_H_38_O_20_ | [M+H]^+^ | 743.2026 | 743.2029 | -0.4 | 465.0919,303.0525,270.1073 | Flavonoids |
| 35 | 11.73 | Peltatoside | C_26_H_28_O_16_ | [M+H]^+^ | 597.1448 | 597.145 | -0.33 | 465.1032,303.0498,115.0393,97.0289,73.0290 | Flavonoids |
| 36 | 12.25 | Cycloolivil | C_20_H_24_O_7_ | [M-H]^-^ | 375.1451 | 375.1449 | 0.53 | 195.0655,179.0704 | Lignans |
| 37 | 12.25 | Quercetin3-O-neohesperi⁃doside | C_27_H_30_O_16_ | [M-H]^-^ | 609.1465 | 609.1461 | 0.66 | 300.0273 | Flavonoids |
| 38 | 13.04 | Rutin* | C_27_H_30_O_16_ | [M-H]^-^ | 609.1467 | 609.1461 | 0.98 | 301.0355,271.0248,151.0025 | Flavonoids |
| 39 | 13.05 | Hyperoside* | C_21_H_20_O_12_ | [M+H]^+^ | 465.1019 | 465.1027 | -1.72 | 303.0499,145.0496,85.0290 | Flavonoids |
| 40 | 13.17 | Liriodendrin | C_34_H_46_O_18_ | [M+HCOO]^-^ | 787.267 | 787.2666 | 0.51 | 579.2069,417.1557 | Lignans |

**Table S2. Cont.**

| **No.** | **RT**  **（min）** | **Components** | **Formula** | **Ion Mode** | **Measured**  **m/z** | **Calculated**  **m/z** | **Mass Error**  **/ppm** | **MS/MS** | | **Class** | |
| --- | --- | --- | --- | --- | --- | --- | --- | --- | --- | --- | --- |
| 41 | 13.29 | Quercetin-3-O-β-D-glucoside | C_21_H_20_O_12_ | [M-H]^-^ | 463.0885 | 463.0881 | 0.86 | 300.0274,271.0250,255.0300,178.9976,151.0026 | | Flavonoids | |
| 42 | 13.79 | Quercitrin | C_21_H_20_O_11_ | [M+H]^+^ | 449.1079 | 449.1078 | 0.22 | 287.0549 | Flavonoids | |  |
| 43 | 13.96 | Reynoutrin | C_20_H_18_O_11_ | [M-H]^-^ | 433.0786 | 433.0776 | 2.31 | 433.0778,301.0353,300.0275 | Flavonoids | |  |
| 44 | 14.17 | Kaempferol3-sambubioside | C_26_H_28_O_15_ | [M-H]^-^ | 579.136 | 579.1355 | 0.86 | 284.0326 | Flavonoids | |  |
| 45 | 14.39 | Quercetin3-O-malonylglu⁃coside | C_24_H_22_O_15_ | [M+H]^+^ | 551.1026 | 551.1031 | -0.91 | 303.0496,231.0498,159.0287,145.0495,127.0391,85.0289 | Flavonoids | |  |
| 46 | 14.72 | Kaempferol-3-O-rutinoside | C_27_H_30_O_15_ | [M-H]^-^ | 593.1517 | 593.1511 | 1.01 | 285.0406,255.0283 | Flavonoids | |  |
| 47 | 15.3 | Naringenin-7-O-glucoside | C_21_H_22_O_10_ | [M^-^H]^-^ | 433.1148 | 433.114 | 1.85 | 271.0614 | Flavonoids | |  |
| 48 | 15.36 | Luteoloside* | C_21_H_20_O_11_ | [M+H]^+^ | 449.1079 | 449.1078 | 0.22 | 287.0549 | Flavonoids | |  |
| 49 | 15.38 | Eucommiol | C_9_H_16_O_4_ | [M-H]^-^ | 187.0967 | 187.0975 | -4.28 | 1690860125 | Iridoids | |  |
| 50 | 15.67 | Quercetin-3-O-glucosyl-6''-acetate | C_23_H_22_O_13_ | [M-H]^+^ | 507.1145 | 507.1133 | 2.37 | 303.0499,187.0603,127.0392,109.0289 | Flavonoids | |  |
| 51 | 15.78 | Kaempferol-3-O-arabino⁃side | C_20_H_18_O_10_ | [M+H]^+^ | 419.097 | 419.0972 | -0.48 | 287.0551,73.0291 | Flavonoids | |  |
| 52 | 15.84 | TortosideA | C_28_H_36_O_13_ | [M-H]^-^ | 579.2084 | 579.2083 | 0.17 | 417.1577 | Lignans | |  |
| 53 | 16.23 | Luteolin7-(6''-malonyl⁃glucoside) | C_24_H_22_O_14_ | [M+H]^+^ | 535.1098 | 535.1082 | 2.99 | 287.0548,231.0396,159.0288,127.0391 | Flavonoids | |  |
| 54 | 16.38 | AIsochlorogenic acidA | C_25_H_24_O_12_ | [M-H]^-^ | 515.1208 | 515.1195 | 2.52 | 353.0879,191.0545,179.0342 | Phenylpropanoids | |  |
| 55 | 16.68 | Isoquercitrin* | C_21_H_20_O_12_ | [M+H]^+^ | 465.1033 | 465.1027 | 1.29 | 303.0499,145.0497,127.0392,85.0290 | Flavonoids | |  |
| 56 | 17.91 | Eriodictyol | C_15_H_12_O_6_ | [M-H]^+^ | 289.0707 | 289.0706 | 0.35 | 271.0613,179.0344,163.0391,153.0182 | Flavonoids | |  |
| 57 | 18.41 | Quercetin* | C_15_H_10_O_7_ | [M-H]^+^ | 301.0353 | 301.0354 | -0.33 | 273.0404,178.9977,151.0026,121.0281,107.0125 | Flavonoids | |  |
| 58 | 18.49 | Caffeic acid | C_9_H_8_O_4_ | [M-H]^+^ | 179.0341 | 179.0349 | -4.47 | 135.0440,89.0230 | Phenolic acids | |  |
| 59 | 20.18 | Naringenin* | C_15_H_12_O_5_ | [M-H]^+^ | 271.0615 | 271.0611 | 1.48 | 177.0184,151.0027,119.0489 | Flavonoids | |  |

**Table S2. Cont.**

| **No.** | **RT**  **（min）** | **Components** | **Formula** | **Ion Mode** | **Measured**  **m/z** | **Calculated**  **m/z** | **Mass Error**  **/ppm** | **MS/MS** | **Class** |
| --- | --- | --- | --- | --- | --- | --- | --- | --- | --- |
| 60 | 20.81 | Kaempferol | C_15_H_10_O_6_ | [M-H]^+^ | 285.0406 | 285.0405 | 0.35 | 173.6046,151.0019 | Flavonoids |
| 61 | 27.27 | 9S,13R-12-Oxophytodienoic acid | C_18_H_28_O_3_ | [M+H]^+^ | 293.2112 | 293.2111 | 0.34 | 275.2007,203.1433 | Organic acids |

* Identified by comparison with a reference standard.

**Table S3 Effect of EULE on the mean lifespan of *C. elegans*: Percentage increase relative to control.**

| Group | Number | Mean lifespan/(days) | % of Control | *p*-Value |
| --- | --- | --- | --- | --- |
| Control | 155 | 14.99 ± 0.17 a | - | - |
| 1 mg/mL | 158 | 16.29 ± 0.39 b | 8.66 | 0.0084 |
| 2 mg/mL | 156 | 17.20 ± 0.26 c | 14.69 | 0.0003 |
| 4 mg/mL | 154 | 16.78 ± .27 bc | 11.89 | 0.0012 |

Values are expressed as mean ± SD (n = 3). Different letters in the same column indicate significant differences (*p* < 0.05).
